# *Plasmodium vivax* epidemiology in Ethiopia 2000-2020: A systematic review and meta-analysis

**DOI:** 10.1371/journal.pntd.0009781

**Published:** 2021-09-15

**Authors:** Tsige Ketema, Ketema Bacha, Kefelegn Getahun, Hernando A. del Portillo, Quique Bassat

**Affiliations:** 1 Jimma University, College of Natural Sciences, Department of Biology, Jimma, Ethiopia; 2 ISGlobal, Institute for Global Health, Hospital Clinic-Universitat de Barcelona, Barcelona, Spain; 3 Jimma University, College of Social Sciences and Humanity, Department of Geography and Environmental Studies, Jimma, Ethiopia; 4 IGTP, Germans Trias i Pujol Health Research Institute, Badalona, Spain; 5 ICREA, Catalan Institution for Research and Advanced Studies, Barcelona, Spain; Kenya Agricultural and Livestock Research Organization, KENYA

## Abstract

**Background:**

Ethiopia is one of the scarce African countries where *Plasmodium vivax* and *P*. *falciparum* co-exist. There has been no attempt to derive a robust prevalence estimate of *P*. *vivax* in the country although a clear understanding of the epidemiology of this parasite is essential for informed decisions. This systematic review and meta-analysis, therefore, is aimed to synthesize the available evidences on the distribution of *P*. *vivax* infection by different locations/regions, study years, eco-epidemiological zones, and study settings in Ethiopia.

**Methods:**

This study was conducted in accordance with Preferred Reposting Items for Systematic Reviews and Meta Analyses (PRISMA) guidelines. Studies conducted and published over the last two decades (2000 to 2020) that reported an estimate of *P*. *vivax* prevalence in Ethiopia were included. The Cochrane Q (χ^2^) and the I^2^ tests were used to assess heterogeneity, and the funnel plot and Egger’s test were used to examine publication bias. A p-value of the χ^2^ test <0.05 and an I^2^ value >75% were considered presence of considerable heterogeneity. Random effect models were used to obtain pooled estimate of *P*. *vivax* infection prevalence. This study is registered with PROSPERO (International Prospective Register of Systematic Reviews): ID CRD42020201761.

**Results:**

We screened 4,932 records and included 79 studies that enrolled 1,676,659 confirmed malaria cases, from which 548,214 (32.69%) were *P*. *vivax* infections and 1,116,581 (66.59%) were due to *P*. *falciparum*. The rest were due to mixed infections. The pooled estimate of *P*. *vivax* prevalence rate was 8.93% (95% CI: 7.98–9.88%) with significant heterogeneity (*I*^2^ = 100%, p<0.0001). Regional differences showed significant effects (p<0.0001, and *I*^*2*^ = 99.4%) on the pooled prevalence of *P*. *vivax*, while study years (before and after the scaling up of interventional activities) did not show significant differences (p = 0.9, *I*^*2*^ = 0%). Eco-epidemiological zones considered in the analysis did show a significant statistical effect (p<0.001, *I*^2^ = 78.5%) on the overall pooled estimate prevalence. Also, the study setting showed significant differences (p = 0.001, and *I*^*2*^ = 90.3%) on the overall prevalence, where significant reduction of *P*. *vivax* prevalence (4.67%, 95%CI: 1.41–7.93%, p<0.0001) was observed in studies conducted at the community level. The studies included in the review demonstrated lack of publication bias qualitatively (symmetrical funnel plot) and quantitatively [Egger’s test (coefficient) = -2.97, 95% CI: -15.06–9.13, p = 0.62].

**Conclusion:**

The estimated prevalence of *P*. *vivax* malaria in Ethiopia was 8.93% with *P*. *vivax* prevailing in the central west region of Ethiopia, but steadily extending to the western part of the country. Its distribution across the nation varies according to geographical location, study setting and study years.

## Author summary

*Plasmodium vivax* is the most widely distributed parasite worldwide. But it is a rare malaria parasite in Africa, except in the eastern part of the region. Ethiopia is one of the few countries in Africa where the two principal human malaria parasite, *P*. *falciparum* and *P*. *vivax* co-exist. Finding of the current review showed that a pooled estimate prevalence of *P*. *vivax* was 8.93% with significant heterogeneity. The prevalence was varied across different regions in the country, eco-epidemiological zones and study settings, where the highest prevalence was documented in the South Nations and Nationalities Peoples’ Region, highlands at an altitude of 2000-2500masl and at health facilities, respectively, while study years (before and after the scaling up of malaria interventional activities) didn’t show any effect on the pooled estimate prevalence of *P*. *vivax*. Overall, *P*. *vivax* showed high prevalence in the western central region of the country, but gradually spreading to the far-western part, previously assumed to be free of malaria. The spread of malaria in general and *P*. *vivax* in particular to malaria free regions could have far reaching consequences and calls for periodic surveillance of the disease to curb the potential public health risks.

## Introduction

*Plasmodium vivax* is one of the five human malaria parasites, with wider distribution across the globe [1]. It causes recurring malaria and affects a large number of populations globally [2]. Although it is widely accepted that the human *P*. *vivax* parasite has African origins [3], its presence in this continent has been unevenly distributed, and its clinical impacts are considered minor except in Eastern Africa [4]. Indeed, the horn of Africa (Ethiopia, Djibouti, Eritrea, and Somalia), South Sudan and the island of Madagascar seem to be the only countries where *P*. *vivax* is considered endemic and causes significant clinical disease in a stable manner, although reports from many other African countries confirm that the parasite does circulate beyond this region. Such a disparate distribution of clinical disease is probably linked to the higher prevalence in these countries (and its generalized absence in the rest of the continent) of Duffy positive individuals, given that this species is thought to require the Duffy receptor to invade reticulocytes and cause disease [5]. However, for the past decade, the increasing demonstration of *P*. *vivax* associated infections and diseases in Duffy-negative individuals from a variety of West African countries [6, 7] confirm the underlying widespread presence of this species across other malaria-endemic regions of Africa, and the possibility that *P*. *vivax* has evolved to find an alternate ways of infecting the reticulocytes and causing disease [8]. Although this phenomenon is yet not widespread, it could further complicate achieving the current malaria elimination goals in the continent [7].

There are additional important knowledge gaps regarding *P*. *vivax*. The parasite’s biology and its pathophysiology are still poorly understood, compared to that of *P*. *falciparum*. Current understanding of the hypnozoite and its basic biology remains elusive, and this is a critical gap that hampers current therapeutic and diagnostic strategies. Moreover, the early release of gametocytes to the bloodstream from the liver, even prior to the appearance of clinical symptoms, facilitates transmission, and obstructs control of this species. Such challenges significantly hamper current global *P*. *vivax* malarial control efforts, and calls for well-coordinated wider ranging research, surveillance and re-mapping of its global epidemiology [9].

Ethiopia accounts for 6% of the malaria cases globally, and about 12% of the global cases and deaths due to *P*. *vivax* [10]. The country has made significant efforts to control malaria since the introduction of dichlorodiphenyl-trichloroethane (DDT) as insecticide upon which the country based its indoor residual spraying (IRS) strategy back in 1959 [11, 12]. Several attempts have been made to scale up major malaria interventional activities such as the distribution of insecticide treated bed nets (ITN), indoor residual spraying (IRS), and introduction of artemisinin-based combination therapy (ACT) starting from 2005 [13]. As a result of these concerted efforts, in areas with Annual Parasite Incidence (API) of > 100 per 1,000 population (high transmission), significant reductions of API (from 14.3 per 1,000 in 2013 to 6.4 in 2016 per 1,000 population) were documented [14]. However, in low transmission areas, the API appeared to increase from 22.5 to 37.4 per 1000 population from 2013 to 2016 [14].

In Ethiopia, where the burden of *P*. *vivax* seems to be slowly rivalling that of *P*. *falciparum*, no attempt has been made to derive a robust epidemiological review of the *P*. *vivax* data available in the country. Clear understanding of the distribution of *P*. *vivax* is essential for informed decisions on appropriate control strategies to be designed and implemented against this neglected species. Thus, the main aim of this review was to synthesize evidence on distribution of *P*. *vivax* infection among symptomatic and asymptomatic cases in Ethiopia.

## Methods

### Research design

The study was conducted according to Preferred Reposting Items for Systematic Reviews and Meta Analyses (PRISMA) guidelines. The protocol was registered at PROSPERO International prospective register of systematic reviews, with ID: CRD42020201761 (available at: https://www.crd.york.ac.uk/PROSPERO/display_record.php?RecordID=201761).

### Search strategy

**Fig 1:**
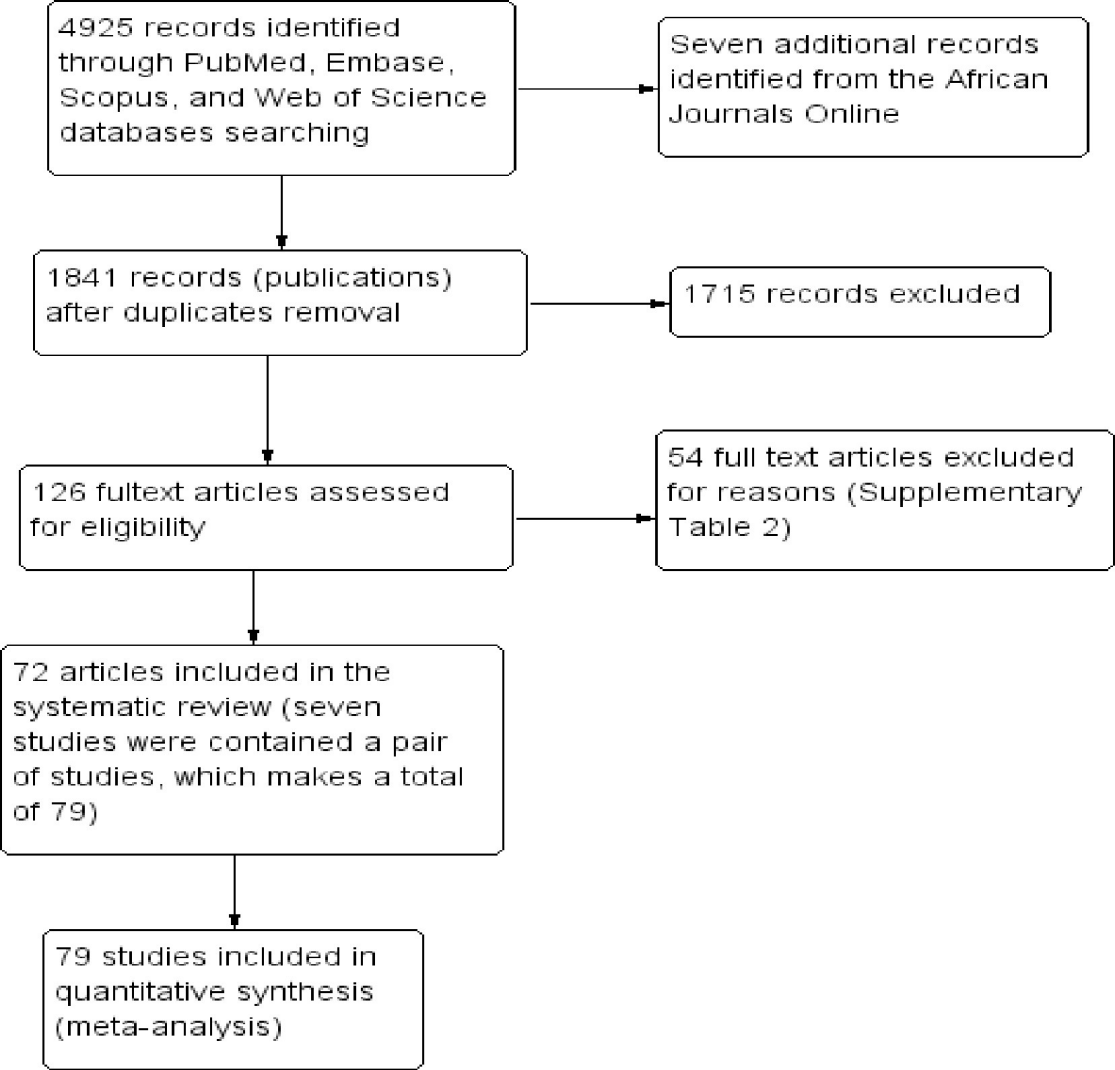
Study flow diagram.

Potentially relevant articles were identified from PubMed (n = 1021), Embase (n = 1250), Web of Science (Core Collection) (n = 1356) and Scopus (n = 1298) electronic databases (Fig 1). A full search strategy for each database was developed using MeSH and free-text words to capture articles measuring *P*. *vivax* prevalence in Ethiopia in human without language restriction (**see S1 Table** for the full detailed search strategies). Each search strategy was applied to articles published between 2000 and 2020. The last search was performed on 31^st^ December 2020. In addition, an effort was made to retrieve more information manually from African Journal Online (AJOL) indexed journals (n = 7). Grey literature and non-published data were not included in the review. Results from different database searches were exported to EndNote and then combined followed by trimming out of any duplicated data.

### Eligibility criteria

Studies were eligible for inclusion if they were original publications describing the epidemiology of *P*. *vivax* in humans in Ethiopia. We included observational studies (cross-sectional and retrospective) written in any language and published over the last twenty years (from 1^st^ January 2000 to December 31st 2020). Studies conducted both in health facilities (i.e., health posts, health centers, and hospitals) and at the community level (i.e., villages, and schools) were included. Other data sources such as reviews, conference abstracts, commentaries, editorials, registered protocols for clinical trials, letters to the editor, personal opinions, non-human or in vitro studies, studies on other *Plasmodium* species and those with incomplete information (studies lacking data on prevalence of *P*. *vivax*) were excluded.

### Study selection

Two authors (TK and KB) independently screened titles and abstracts of all records identified by the search strategy for potential inclusion in the review. Afterwards, full-text copies of articles deemed potentially relevant were retrieved and their eligibility was assessed. Disagreements between individual judgments were resolved through discussion. We listed all studies excluded after full-text assessment and reasons for the exclusion **(S2 Table).**

### Data extraction

Two authors (TK and KB) used a data extraction form to independently extract data on study characteristics, including: type of study (facility or community based), age group, and presence or absence of symptoms. Additional information collected included study year (before or after the scale up of national malaria interventional activities) [14], geographical regions, diagnostic methods used, sample size, and the main characteristics of the population under study.

*Outcome of interest was p*revalence of *P*. *vivax* infection. *P*. *vivax* malaria diagnosis required parasitological confirmation irrespective of the methods used (optic microscopy, RDT, PCR, LAMP, ELISA, etc.). Original authors were contacted when further clarification and additional data were necessary.

### Assessment of risk of bias in included studies

The risk of bias for each included study was assessed independently by two authors (TK and KB) using the Prevalence Critical Appraisal Instrument, designed to be used in systematic reviews addressing questions of prevalence, as described by Munn et al. [15]. This tool assesses the methodological quality of studies reporting prevalence data using ten critical appraisal criteria: sample representation of the target population, participant recruitment appropriateness, sample size adequacy, subjects and setting detailed description, enough coverage of the identified sample, objectivity and standardization in the measurement of the condition, reliability in the measurement of the condition, statistical analysis appropriateness, confounders/ subgroups/differences identification and accounting, and subpopulations identification using objective criteria. An overall low (≥7/10), medium (between 5 and 7/10), high (<5/10) risk of bias level was assigned to each study.

### Data synthesis and analysis

Data were analyzed using the Cochrane Review Manager (version 5.4) for qualitative and quantitative synthesis. Prevalence for each study was reported. For cases where prevalence was not reported, authors calculated it by dividing the event (*P*. *vivax* positive and/or in mixed infection) to the total population sampled in each study. Standard error of the mean (SE) for each study was calculated from the standard deviation obtained using the formula, $\mathrm{StDev}=\sqrt{p(1-p)}$ where **p** is a proportion of the population with the event. Then, SE was calculated from the *StDev* using the formula, $\mathrm{SE}=\mathrm{StDev}\sqrt{n}$, where n is the sample size.

Heterogeneity between studies was evaluated using Cochrane’s Q (χ^2^) and the I^2^ tests. For the Cochrane’s test, a p-value of the χ^2^ test less than 0.05 was considered as significant statistical heterogeneity. I^2^ values of 25%, 50% and 75% were assumed to represent low, medium, and high heterogeneity, respectively. Outliers that might cause heterogeneity and meta-coefficient were analyzed using Comprehensive Meta-analysis (CMA) software and presented using box plots (**S1 Fig**) and Table, respectively.

Subgroup analysis was conducted to investigate heterogeneity. Pre-specified subgroups potentially assumed to affect the overall prevalence estimate included: i) geographical location/regions (in Ethiopia there are currently ten regional states and two chartered cities), ii) study setting, iii) eco-epidemiological zones (altitude), and iv) study year. Likewise, due to high heterogeneity (I^2^ > 75%, P < 0.05), random effects models were used for the pooled statistics. Forest plots were used to display point estimates and confidence intervals. Publication bias for studies included in the meta-analysis was assessed quantitatively using the Egger’s test and qualitatively constructing funnels plot and looking for asymmetry. ArcGIS software version 10.0 was used to sketch a map for the distribution of *P*. *vivax* malaria in the country.

## Results

### Study selection

A total of 4932 citations were initially identified. After the duplicates were excluded, 1841 unique citations were screened and assessed for eligibility. From the remaining 1841 screened at title/abstract level, 1715 records considered irrelevant for the purposes of the study were excluded. At the second phase of records assessment, a total of 126 eligible studies with available full text were thoroughly reviewed and a total of 72 articles (seven of them were comprised of a pair of an independent studies, which makes the total of studies 79) included for qualitative and quantitative meta-analysis, respectively (Fig 1). Detailed reasons for the 54 excluded studies are presented in **S1 Table.**

### Quality assessment of individual studies

Across the 10 quality domains evaluated, the majority of the studies met five or more of the quality criteria. Most of the studies (n = 31) met 8 or more of the quality criteria assessed, and others (n = 26) met 5 to 7 of the quality criteria assessed for prevalence studies. Only 15 studies were rated below 5 for the quality assessment. The most common quality criteria not fulfilled by the studies were: poor statistical analysis such as failure to use reliable, valid and appropriate data analysis tools (n = 27), failure to identify confounders/differences accounting (n = 24) and unclear sample recruitment (n = 19). Most of the studies fulfilled the following quality criteria: contained adequate sample size (n = 64), described the study subjects and setting in detail (n = 62), and the data analyses were conducted with sufficient coverage of the identified samples (n = 69). Nine studies met all 10 quality assessment criteria. Twenty-eight studies were based on data extracted from patients’ medical records accessed from health facilities. For such studies, some of the quality criteria such as defining target population, use of appropriate sampling techniques and standard data collection tools/methods were difficult to evaluate and were considered as not applicable (NA) (**S3 Table**).

### Study characteristics

**Fig 2:**
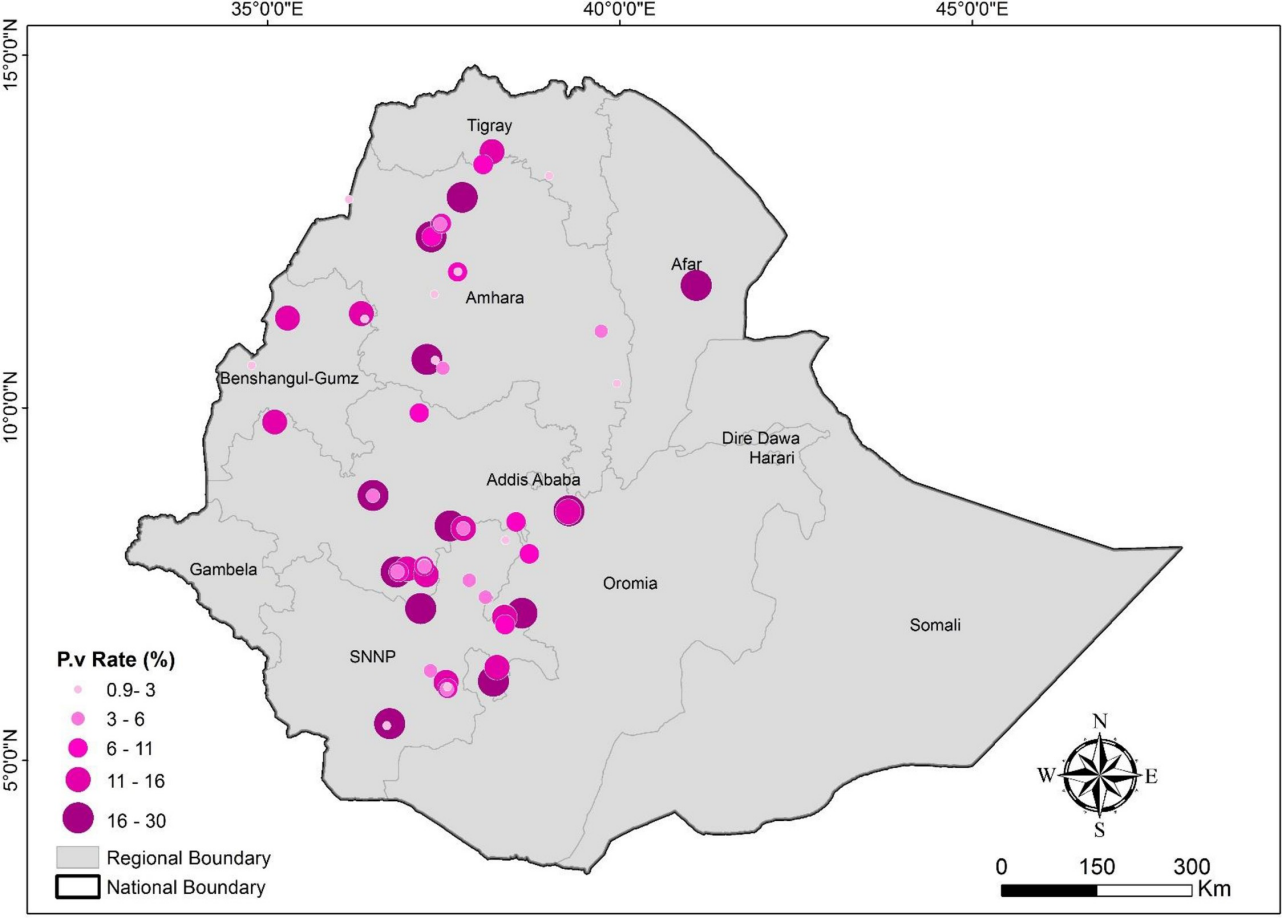
Map showing estimates of *P*. *vivax* prevalence from the 72 study sites according to geographical distribution in Ethiopia. The size of the purple dots is proportional to the prevalence estimates reported. The map was sketched by one of the authors using ArcGIS software.

A total of 72 articles, but 79 studies, were finally included in the meta-data analysis, 18 studies have reported data from 8 study sites (more than one study from single site), at different years and seasons, and by different authors using different study populations. They reported on prevalence data from the following towns: **Arbaminch** [16–18], **Arijo Didhesa** [19, 20], **West Armachew** [21, 22], **Butajira** [23, 24], **Dore Bafeno** [25, 26], **Jimma town** [27, 28], **Wolkite** [29, 30], and **Woreta** [31, 32]. The rest of the studies typically reported data from a single study site, although some reported data for multiple seasons (Fig 2).

Twenty-eight studies reported pooled prevalence data based on retrospective evaluations of 5–20 years’ patient data collected from health facilities. The remaining 51 were cross sectional studies undertaken at health facilities (n = 60) or at the community level (n = 19). Malaria diagnosis relied on optic microscopy in the majority of studies (n = 60/79, 75.95%); with the remaining 19 studies using either only RDT (n = 3), microscopy plus RDT (n = 11), microscopy plus PCR (n = 2), a mix of the three techniques (microscopy, RDT and PCR; n = 3). Participants of most of the included studies (n = 59/79, 74.7%) were all-age groups populations, while 11 were from children and teenagers up to 15 years of age, five studies included population aged >15 years and four studies enrolled only pregnant women. The 79 studies enrolled a total of 5,930,976 study participants (ranging from 178 to 2,827,722) among which 1,676, 659 were malaria positive. A total of 548,214 participants [about 9.24%, (ranging from 1 to 267,242)] had a confirmed *P*. *vivax* infection [mono infection (n = 525,674; 95.9%) and mixed infection (n = 22,406; 4.1%)] [16–86]. Ethiopia is a federal state https://en.wikipedia.org/wiki/Federation subdivided into ethno-linguistically based regional states. There are currently ten regional states and two chartered cities. In line with this division, the studies reported data from the regions of Afar (n = 1), Amhara (n = 26), Benishangul (n = 3), Oromia (n = 18), Southern Nations, Nationalities and Peoples’ Region (SNNPR) (n = 25), Tigray (n = 1), Harari (n = 1) and nationwide surveys of Ethiopia (n = 4). Accordingly, the majority of the malaria research reports (69/79, 87.34%) presented data from Amhara, Oromia and SNNPR. Based on the eco-epidemiological zones of malaria distribution, 22 studies were reported from areas with altitude <1500m (low lands with seasonal/intense transmission), 10 were from altitudes between 1500-1750m (high land fringe, high unstable transmission), 14 were from altitudes ranging between 1750-2000m (high land fringe, low unstable transmission), 7 studies were from districts with altitudes of 2000-2500m (highland, occasional epidemic) and 23 were from areas with mixed ecological zones (Table 1), and three studies without this information were excluded [48, 49].

**Table 1:** Characteristics of the studies included in the epidemiological studies of *P*. *vivax* in Ethiopia (2000–2020).

Author ID	Study site/ City/district	Region	Altitude (m)	Setting	Study design	Study year/period	Sample tested	Study population			Diagnostic method	Malaria positive	*P*. *falciparum*	*P*. *vivax*	Mixed infection	Group
								Key characteristics	Age	Gender						
Abossie et al., 2020	Arbaminch	SNNPR	1,285	Health facility	Cross-sectional	April 2017—May 2017	271	Febrile children. Exclusion if antimalarial drug administration up to 3 months prior to the study	Range: 12–59 months; Mean: 31.2 months	58% males, 42% females	Microscopy	60	30	29	1	Children
Addisu et al., 2020	Gorgora and Chuahit in Dembia district.	Amhara	1, 850–2, 000	2 health facilities	Retrospective clinical record review	2012–2018	11,879	Patients that were requested a blood film	All ages	57% males, 43% females	Microscopy	2590	1756	733	101	All ages
Alelign et al., 2018	Woreta town, Fogera district	Amhara	1828	Health facility	Retrospective clinical record review	2005–2012	102,520	Suspected cases of malaria	All ages	53% males, 47% females	Microscopy	33431	23274	8870	1287	All ages
Alemayehu et al., 2015	Diverse	Oromia	Mix	12 health facilities	Cross-sectional	Sept 2011—Nov 2011	1,819	HIV-positive patients having routine follow-up visits at HIV care and treatment clinics	≥ 18 years	36% males, 64% females	Microscopy	13	6	7	ND	≥ 18 years
							1,819	HIV-sero-negative patients attending the general medical outpatients departments	≥ 18 years	54% males, 46% females	Microscopy	143	69	74	ND	≥ 18 years
Alemu & Mama, 2018	Arbaminch	SNNPR	1,285	Blood bank	Cross-sectional	Feb 2015—June 2015	416	Blood donors, asymptomatic. Exclusion of permanent residents of known non-endemic malaria areas	Range:18–59 years; Median: 22 years	56% males, 44% females	Microscopy	17	8	9	ND	≥ 18 years
Alemu et al., 2011	Jimma town	Oromia	1,750	Community, house-hold-based survey	Cross-sectional	April 2010—May 2010	804	Households’ residents	All ages; Median: 21 (SD 1.2) years	42% males, 58% females	Microscopy	42	11	30	1	All ages
Alemu et al., 2012b	Azezo	Amhara	1,400	Health facility	Cross-sectional	Feb 2011—March 2011	384	Febrile patients. Exclusion of pregnant women, if known concomitant chronic infections, or if antimalarial drug administration in the 2 weeks prior to the study	Range: 1–80 years; Median: 23.8 years	51% males, 49% females	Microscopy	44	9	33	2	All ages
Alemu et al., 2014	Dabat district	Amhara	Mix	4 health facilities	Cross-sectional	August 2012—May 2013	1,644	Residents visiting local health centers	All ages	ND	Microscopy or RDT	645	355	173	117	All ages
Alkadir et al., 2020	Mankush	Benshangul	ND	Health facility	Retrospective clinical records review	Jan 2014—Dec 2018	16,964	Malaria suspects	All ages	ND	Microscopy	8658	6513	2121	24	All ages
Animut et al., 2009	Dembecha, Jiga, Gebeze Mariam, Finoteselam	Amhara	ND	4 health facilities	Cross-sectional	Sep 2006—Nov 2006	653	Febrile outpatients. Exclusion of children requiring inpatient treatment or with chronic disease	Range: 3–17 years; Median: 8.4 years	51% males, 49% females	Microscopy	506	309	150	47	All ages
Argaw et al. 2016	Diverse	Mix	Mix	110 health facilities	Retrospective clinical records review	April 2012—Sep 2015	873,707	Malaria suspected patients with a diagnostic test result	All ages	60% males, 40% females	Microscopy and RDT	223,293	108704	96765	8790	All ages
Aschale et al., 2018	West Armachiho district	Amhara	667	Community, 10 farm sites	Cross-sectional	Sep 2016—Dec 2016	385	Asymptomatic migrant laborers	Range: 15–60 years; Mean: 26.3 (SD 8.9) years	90% males, 10% females	Microscopy	71	50	7	14	≥15 years
Aschale et al., 2019	West Armachiho district	Amhara	667	Community, 11 farm sites	Cross-sectional	Oct 2016—Dec 2016	178	Migrant laborers. Exclusion if taken medication for malaria and/or visceral leishmaniasis for the last 2 weeks	Range: 15–65 years; Mean 26.1 (SD 8.6) years	92% males, 8% females	Microscopy	40	29	4	7	≥15 years
Ashton et al. 2011	Diverse	Oromia	Mix	Community, school-based survey (197 schools)	Cross-sectional	May 2009, Oct 2009-Dec 2009	20,899	Children. Excluded if the blood film was missing or unreadable	Range: 5–18 years; Median 11 (IQR: 9–12).	53% males, 47% females	Microscopy^1^	117	61	55	1	Children
Assefa et al., 2015	Hossana	SNNPR	2,177	Health facility	Cross-sectional prior to an RCT	April 2014	1,693	Clinically malaria-suspected individuals with fever or history of fever seeking treatment	All ages	ND	Microscopy	281	182	92	7	All ages
Awoke & Arota, 2019	Tercha Hospital	SNNPR	1406	Facility	Cross-sectional	March 20 to May 30, 2016.	340	All acute febrile patients clinically suspected of malaria	Range: 15–50 years; Mean 27.6	68% males, 32% females	Microscopy	170	105	61	4	All ages
Ayalew et al., 2016	Jiga area	Amhara	1,812	Community, household-based survey	Cross-sectional	Nov 2013—Dec 2013	392	Households’ residents (one person randomly selected per household)	Range: 1–80 years; Mean 21.9	38% males, 62% females: 9% self-reported pregnant	RDT	11^2^	6	5	0	All age
Belete and Roro., 2016	Chichu, Wonago	SNNPR	1,650	Health facility	Cross-sectional	May 2016—June 2016	324	Outpatients with history of fever in the last 24h. Exclusion if not resident or anti-malarial treatment during the previous 8 days	All ages	53% males, 47% females	Microscopy	91	32	48	11	All ages
Birhanie et al., 2014	Dembia district	Amhara	1,750–2,100	Health facility	Cross-sectional	April 2013—May 2013	200	Febrile patients suspected for malaria and/or typhoid fever. Exclusion if antimalarial treatment and/or antibiotics within the previous 2 weeks	Range: 2–80 years; Mean 24.2 (SD: 13.4)	60% males, 40% females	Microscopy	73	32	30	11	All age
Beyene et al., 2020	Jardga Jarete district	Oromia	1,400–2,700	3 health facilities	Retrospective clinical records review	2015–2019	25,868	Malaria suspects. Excluded if malaria diagnosis results were not properly documented	≥ 1 year	60% males, 40% females	Microscopy	4,336	2,561	1434	342	All age
Dabaro et al., 2020	Boricha district	SNNPR	1001–2076	51 Health facilities	Retrospective clinical records review	2010–2017	135,607	Malaria suspects. Exclusion if incomplete record	All ages	51.4% males, 48.4% females	Microscopy or RDT	29,554	16,647	11,360	1,547	All ages
Debo & Kassa, 2016	Benna Tsemay district	SNNPR	1,500	Community, household-based survey	Cross-sectional	Dec 2011—Jan 2012	461	Household residents of pastoralist communities	Range: 9 months– 65 years; Median: 13 years	48% men, 52% female (7% pregnant,7.5%lac tating)	Microscopy or RDT	28	18	6	4	All ages
Degarege et al., 2011	Dore Bafeno	SNNPR	1,708	Health facility	Cross-sectional	January, 2010	269	Malaria suspects. Exclusion if anti-malarial treatment within the previous 2 weeks	All ages	53.5% males, 46.47% females	Microscopy	178	146	28	4	All ages
Degarege et al., 2012	Dore Bafeno	SNNPR	1,708	Health facility	Cross-sectional	Dec 2010—Feb 2011	1,065	Malaria suspects. Exclusion if anti-malarial treatment within the previous 2 weeks	Range: 1–82 years; Mean 18.6 years	51% males, 49% females	Microscopy	306	138	154	14	All ages
Delil et al., 2016	Hadiya zone	SNNPR	2,106	12 health facilities	Cross-sectional	May- June, 2014.	411	Febrile patients	Range: 18 years to 70 years, Mean 30.7 years	50.4% males, 49.6% females	Microscopy	106	27	76	3	Adult >18
Demissie and Ketema, 2016	Mendi	Oromia	1,538	2 health facilities	Cross-sectional	Sep 2014—June 2015	4,813	Malaria suspects	Range: one month- 60years, median age 14 years	ND	Microscopy	1,434	851	533	50	All ages
Derbie and Alemu, 2017	Woreta	Amhara	1,828	Health facility	Retrospective clinical records review	Sep 2011—August 2012	8,057	Malaria suspects. Exclusion if incomplete record	Range: 1–85 years; Median 25 years	45% males, 55% females	Microscopy	435	233	184	17	All ages
Dufera et al., 2020	Arjo Didhessa sugar cane plantation area	Oromia	1275–1570	Community, household-based survey	Cross-sectional	May 2016—Nov 2017	443	Household’s residents	All ages	ND	Microscopy	14	6	8	ND	All ages
				Health facility	Retrospective clinical records review	2013–2017	65,275	Outpatients	All ages	ND	Microscopy	4,164	776	3,170	218	All ages
Ergete et al., 2018	Salamago and Benatsemay districts	SNNPR	Mix	2 health facilities	Retrospective clinical records review	Jan 2008—Dec 2014	54,160	Malaria suspects with a blood smear	All ages	61% males, 39% females	Microscopy	22,494	13,727	7,297	1,470	All ages
Esayas et al., 2020a	Kolla-Shara village	SNNPR	1,170–1,390	Community, household-based survey	Prospective (repeated cross-sectionals)	July 2016—Dec 2016	131	Febrile household’s residents. Individuals were screened twice per month for fever episodes	All ages	ND	RDT and microscopy confirmation	46	27	19	ND	All ages
Esayas et al., 2020b	Harari	Harari	1552–1957	Health facility	Retrospective clinical records review	2013–2019	95,629	Malaria suspected cases	All ages	ND	Microscopy or RDT	44,882	28,576	12576	77	All ages
Feleke et al., 2018	Ataye	Amhara	1,468	Health facility	Retrospective clinical records review	2013–2017	31,810	Malaria suspects. Exclusion if record incomplete	All ages	ND	Microscopy	2,670	2,087	557	26	All ages
Feleke et al., 2020	North-Shoa zone	Amhara	1,532–1,788	3 health facilities	Cross-sectional	Nov 2018—Jan 2019	263	Asymptomatic pregnant women. Exclusion if disease symptom/signs within the last 48h, treated with anti-malarial drugs in the previous 6 weeks, long-term medical treatment uptake or non-permanent resident in the area	Range: 16–41 years; Mean 27.8 (SD: 5.3) years	-	Microscopy^3^	15	9	6	0	Pregnant
Ferede et al., 2013	Metema	Amhara	685	Health facility	Retrospective clinical records review	Sep 2006—Aug 2012	55,833	Malaria suspects	All ages	54% males, 46% females	Microscopy	9,486	8,602	852	32	All ages
Gebretsadik et al., 2018	Kombolcha	Amhara	1,875	Health facility	Retrospective clinical records review	2009–2016	27,492	Malaria suspects. Exclusion of incomplete records	All ages	ND	Microscopy	2,066	1,243	734	89	All ages
Geleta and Ketema	Pawe district	Benishangul	1050	Health facility	Cross-sectional	October 2013 to May-2014	1523	Malaria suspected cases	All ages	ND	Microscopy	623	420	140	63	All ages
Golassa & White, 2017	Adama malaria diagnostic centre	Oromia	1,712	Health facility	Cross-sectional	May 2015—April 2016	3,161	Malaria suspects	All ages	68% males, 32% females	Microscopy	1,141	326	847	32	All ages
Gontie et al., 2020	Sherkole district	Benishangul	680–800	Community	Cross-sectional	July 2018—August 2018	498	Pregnant women. Exclusion if mental illness or severely debilitating disease	≥ 15 year	-	RDT	51	46	5	ND	Pregnant women
Haile et al., 2020	Dembecha	Amhara	2,083	Health facility	Retrospective clinical records review	Sep 2011—August 2016	12,766	Malaria suspects. Exclusion of incomplete records.	All ages	57% males, 43% female	Microscopy	2,086	1,433	549	104	All ages
Haji et al., 2016	East Shewa zone	Oromia	1,549–2,093	5 health facilities	Cross-sectional	Oct 2012- Nov 2012	830	Malaria suspects	< 16 years; Mean: 6 years; Median: 6.1 years	49% males, 51% females	Microscopy^4^	170	70	97	3	Children
Hassen & Dinka, 2020	Batu town	Oromia	1657	Health facility	Retrospective clinical records review	2012–2017	175423	Malaria suspected cases	All ages	53% males, 47% females	Microscopy	21797	10791	11006	ND	All ages
Hawaria et al., 2018	Arjo-Didessa sugar development site	Oromia	1300–2280	Health facility	Retrospective review clinical records registers of 11 health facilities	2008–2017	54020	Malaria suspected cases	All ages	64.5% males, 35.5% female	Microscopy, RDT	18049	8660	7649	1740	All ages
Ifa, 2018	Konga Health Center	SNNPR	2044	Health facility	Retrospective clinical records review	2011–2015	5210	Malaria suspected cases	Children under five years	51% males, 49% females	Microscopy	2459	1402	1057	ND	Children
Jemal and Ketema, 2019	Asendabo town	Oromia	1791	Health facility	Retrospective clinical records review	2007–2016	68421	Malaria suspected cases	All ages	52.5% Males, 47.5% females	Microscopy	13624	7087	6508	29	All ages
Kalil et al., 2020	Bale zone	Oromia	Mix	Health facility	Retrospective clinical records review	January 2010- December 2017	62,392	malaria suspected individuals who had visited the health facilities in Bale zone	All ages	63% males, 37% females	Microscopy or RDT	10,986	9,850	2036	ND	All ages
Karunamoorthi & Bekele, 2009	Serbo health center, Jimma zone	Oromia	1740–2660	Health facility	Cross-sectional	July 2007 and June 2008	6863	Febrile patients presenting malaria symptoms	All ages	64% males, 36% female	Microscopy	3009	1946	1052	11	All ages
Lankir et al., 2020	Central, North and West Gondar zones	Amhara	Mix	Health facility	Retrospective clinical records review	July 2013–June 2018	2,827,722	Malaria suspected cases	All ages	ND	Microscopy or RDT	1,003,391	736,149	266,797	445	All ages
Legesse et al., 2015	Wolita zone	SNNPR	2950	Health facility	Retrospective clinical records review	2008–2012	317,867	Malaria suspected cases	All ages	51% males, 49% female	Microscopy	105,755	75,927	25,329	4497	>15 years
Lo et al. 2015	Six different localities across Ethiopia (Bure, Halaba, Asendabo, Jimma, Menkusha, Metehara, Shewarobit	Ethiopia	Mix	Community	Cross-sectional	ND	390	Asymptomatic individuals representing the younger age < 18 years and older age >18 years	All ages	ND	Nested PCR of the 18S rRNA region	73	49	23	1	All ages
				Health facility	Cross-sectional	ND	416	Symptomatic or febrile patients visiting the health centres or hospitals	All ages	ND	Nested PCR of the 18S rRNA region	331	134	164	33	All ages
Mekonnen et al., 2014	Omo Nada, Bala Wajo and Arba Minch	Oromia, SNNPR	MiX	Health facility	Cross-sectional	August and December 2011	1416	Self-presenting febrile patients attending health centres	All ages	60.2% males, 39.8% females	Microscopy and PCR	307	125	154	24^5^	All ages
Minwuyelet et al., 2020	Gondar Zuria district	Amhara	1750–2600	Community	Cross-sectional	May- June 2019	251	Individuals with clinical symptom of malaria and those taking antimalarial drugs 1 month prior to data collection excluded	All ages, mean: 24.6 years	47% males, 53% females	Microscopy	30	5	25	ND	All ages
Nega et al., 2015	Arbaminch town	SNNPR	1,200–1,300	Community	Cross-sectional	April and June, 2013	341	Pregnant women without disease symptom/sign within the past 48 hours	ranged from 17 to 40 years with a median age of 25		Microscopy, or RDT	31	12	15	4	Pregnant women
Schicker et al., 2015	Metema and west armachiho	Amhara	717	Community	Cross-sectional	17–26 July, 2013	592	a venue-based survey of 605 migrant laborers 18 years or older	>18 years, mean: 22.8 years	98% males, 2% females	RDT	71	57	10	4	>18 years and above
Shamebo and Petros., 2019	Halaba special district	SNNPR	1554 to 2149	Health facility	Retrospective clinical records review	September 2013- August 2017	583668	Malaria suspect cases	All ages	49.8% males, 50.2% females	Microscopy	55252	21397	33855	ND	All ages
Shiferaw et al. 2018	Tselemti District	Amhara	1400	Health facility	Retrospective clinical records review	January 2013 and December 2015	41773	Malaria suspect cases	All ages	54% males, 46% females	Microscopy	11745	6835	4165	745	All age
Solomon et al., 2020a	Wolkite health center Gurage zone	SNNPR	1910–1935	Health facility	Retrospective clinical records review	January 2015—December 2018	121230	Malaria suspected cases	All ages, majority(54%) were >15 years	51% males, 48.3% females	Microscopy	10379	3044	7239	98	All ages
Solomon et al., 2020(b)	Wolkite health center Gurage zone	SNNPR	1910–1935	Health facility	Cross-sectional	June 2019—August 2019	230	asymptomatic pregnant women	>18 years, majority (72.2%) were between 18–27 years	-	Microscopy	50	20	30	ND	Pregnant women
Tadesse and Tadesse, 2013	Felegeselam Health Center	Amhara	1000–1050	Health facility	Cross-sectional	December, 2011	398	Acute febrile patients	All ages	51% males, 48.2 Females,	Microscopy	201	194	7	ND	All ages
Tadesse et al., 2015	Malo (Salayish Mender 4 and Tatta-qirchiqircho)	SNNPR	591	Community	Cross-sectional	February 2014, in the dry season	555	Asymptomatic Community members residing in the study sites for at least 2 years	All ages		Microscopy, RDT, nested PCR	54	29	24	1	All ages
Tadesse et al., 2017	Andassa, Yinessa, Ahuri, Yeboden, Fendika schools	Amhara	1218–2010	Community: five elementary schools	Cross-sectional	First survey June, 2015	555	Students attending the elementary schools	Children, median age is 12 years	51.3% males, 48.7% females	Microscopy, RDT, 18S based nested PCR, ELISA	56	43	13	ND	Children
						second survey November 2015	294	Students attending the elementary schools	Children, median age is 12 years	51.3% males, 48.7% females	Microscopy, RDT, 18S based nested PCR, ELISA	52	38	14	ND	Children
Tesfa et al., 2018	Adi Arkay health centre	Amhara	1750–2100	Health facility	Retrospective clinical records review	1997–2013	20,483	Malaria suspected cases	All ages	ND	Microscopy	7392	5089	2128	173	All age
Tesfaye et al., 2011	Butajira district	SNNPR	1900	Community	Cross-sectional	October, November, and December, 2006	1082	Members of two farming associations	>15 years old	52% males, 48 females	Microscopy	48	16	32	ND	All ages
Tesfaye et al., 2019	Tanquea Abergelle	Tigray	1542	Community	Cross-sectional	September 8 to October 18, 2017	1300	Malaria suspected cases	All ages	46.6% males, 53.4% females	Microscopy	876	856	20	2	All ages
Tuasha et al., 2019	Kella, Aruma and Busa Health Centers in Wondo Genet	SNNPR	1880	Health facility	Cross-sectional	December 2009 to July 2010	427	malaria suspected febrile patients from three health centers	ranged from 6 -77years (mean ± SD = 20.8 years	55% males, 45% females	Microscopy	276	202	71	3	All ages
Woday et al., 2019	Dubit district	Afar	800–1000	Health facility	Cross-sectional	April 15th to 15th May 2018	484	All under-five children who presented with fever symptoms	Children, mean age was 28 months	56.6% males, 43.4% females	Microscopy or RDT	310	206	72	32	children
Wondimeneh et al., 2018	Kolla-Diba health center	Amhara	2040	Health facility	Cross-sectional	November 01, 2015 to May 30, 2015	384	HIV positive febrile patients	All ages, mean age of 28 years	59% males, 41% females	Microscopy	53	8	4	0	All ages
								HIV negative febrile patients	All ages, mean age of 28 years	59% males, 41% females	Microscopy	79	43	31	5	All ages
Woyessa et al., 2012	Butajira area (six kebeles)	SNNPR	1800–2300	Community	Cross-sectional	October 2008 to June 2010	19,207	all family members who consented to the study	Ranged: 0 months-99years, mean age was 20.5 years	48.7% males, 51.3% females	Microscopy	178	22	154	2	All age
Yehualaw et al., 2009	Gilgel-Gibe hydroelectric dam	Oromia	1,734–1,864	Community	Cross-sectional	October and December 2005	1855	At risk Children those living in villages within 3 km of the reservoir	children under 10 years	48.8% males, 51.2% females	Microscopy	142	59	83	ND	Children
							774	Control, Children living in villages within 5-8km from its shore	children < 10 years, mean age:4.7 years	48.7% males, 51.3% females	Microscopy	51	17	34	ND	Children
Yimer et al., 2015	Walga, Borer, Jeju, and Nacha Qulit kebeles	SNNPR	1100–2300	Community	Cross-sectional	December 2013	400	afebrile individuals residing in the visited house holds	All ages	42% males, 58% females	Microscopy	1	0	0	1	All ages
	Walga Health Center Abeshge District,	SNNPR	1100–2300	Health facility	Retrospective clinical records review	February 2008 and December 2012	34,060	Malaria suspected cases	All ages	52% males, 48% females	Microscopy	11523	5889	5489	150	All ages
Yimer et al., 2017	Felegehiwot referral Hospital	Amhara	1840	Health facility	Retrospective clinical records review	2010–2014	14,750	Malaria suspected cases	All ages	50.3% males, 49.7% females	Microscopy	740	397	331	12	All ages
Zerihun et al., 2011	Dore Bafeno Health Center,	SNNPR	1708	Health facility	Cross-sectional	January 2010.	269	febrile outpatients who sought medical attention	All ages	53% males, 47% females	Microscopy	178	146	28	4	All ages
Zhou et al., 2016	Jimma town	Oromia	1710–1800	Health facility	Cross-sectional	July 2014 to June 2015	1434	Malaria suspected cases	ND	48% males, 52% females	Microscopy	428	327	97	4	All ages

*Note*: *ND =* No data available; SNNPR = Southern Nation and Nationalities People Region; RDT = Rapid Diagnostic Test; PCR = Polymerase Chain Reaction; M = Male, F = Female, Mixed infection: *P*.*falciparum* and *P*. *vivax* infection

^1^ RDT was also performed in a subset of individuals. Discrepant results between microscopy and RDT were solved by a second microcopy reading

^2^ Crude results, not results weighted for HH size

^3^ RDT was also performed, but species information is only based on microscopy

^4^Except 2 tests in which RDTs were used

^5^ Mixed infections: *P*. *falciparum* and *P*.*vivax* (n = 24), and *P*. *falciparum* and *P*. *malariae* (n = 4)

### Main outcome of the meta-analysis

**Fig 3:**
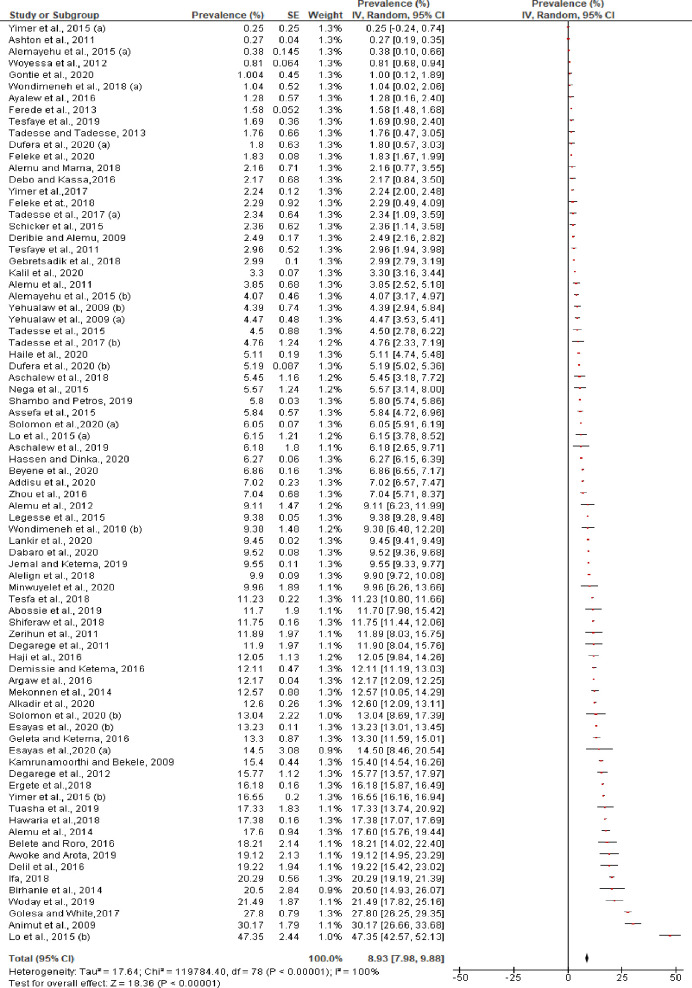
Individual and pooled estimates of the prevalence of *P*. *vivax* (mono-infection and mixed infection with *P*. *falciparum*) in Ethiopia.

The overall random effects pooled prevalence rate of *P*. *vivax* (mono-infection and mixed infection with *P*. *falciparum*) in Ethiopia was 8.93% (95% CI: 7.98–9.88%), with a very high level of heterogeneity (*I*^2^ = 100%, p<0.0001). Indeed, the prevalence of *P*. *vivax* across individual studies varied considerably [ranging from 0.25, n = 1/400 among all age groups in SNNPR [85] to 47.35%, n = 197/416 in all age groups in many sites throughout Ethiopia using 18r based nested PCR [74] (Fig 3).

**Fig 4:**
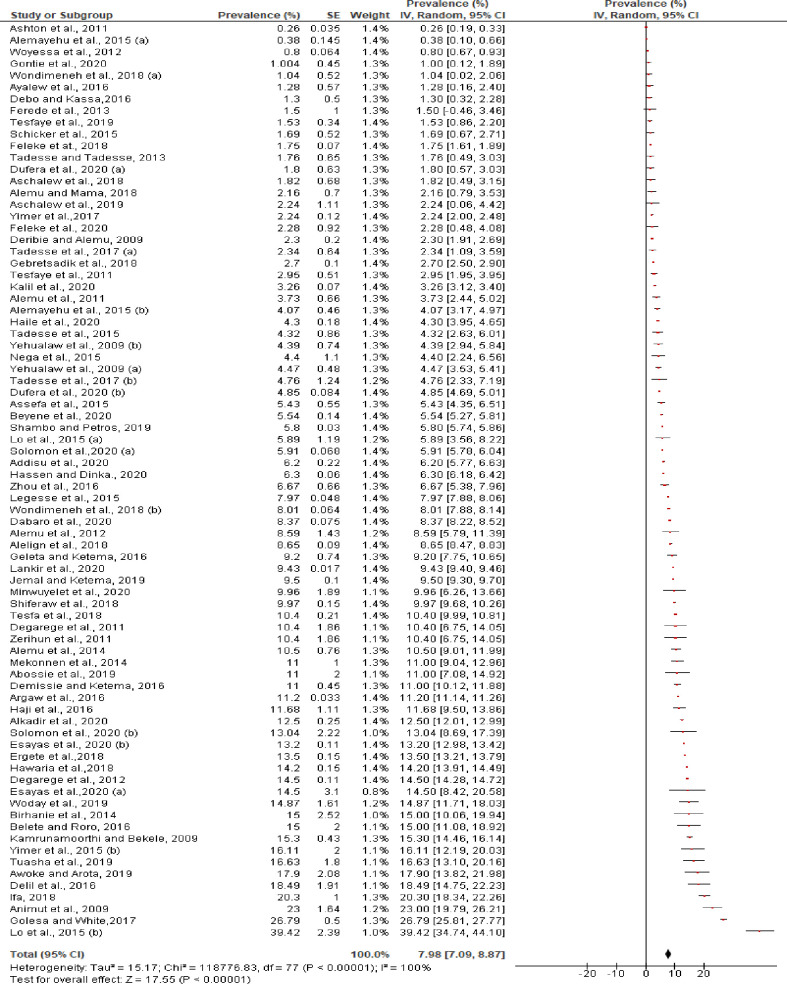
Individual and pooled estimates of the prevalence of *P*. *vivax* mono-infection in Ethiopia, 2000–2020.

**Fig 5:**
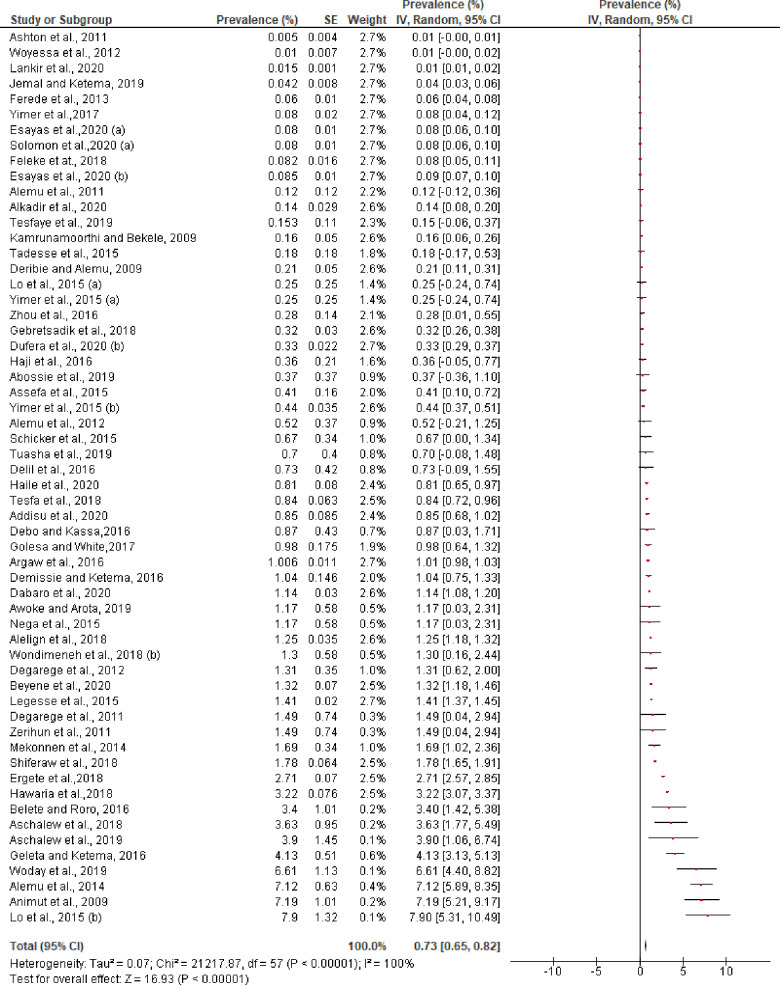
Individual and pooled estimates of the prevalence of mixed infection (*P*. *vivax* and *P*. *falciparum*) in Ethiopia, 2000–2020.

The pooled prevalence of *P*. *vivax* in mono-infection was 7.98% (95% CI: 7.09–8.87%) with a very high level of heterogeneity (Fig 4) and prevalence of *P*. *vivax* in a mixed infection (*P*. *vivax* with *P*. *falciparum)* was 0.73% (95% CI: 0.65–0.82%). The prevalence reported in each study for mixed infection was also varied and ranged from 0.005% [51] to 7.9% [74] (Fig 5). Analysis of risk of publication bias among the studies included in the current review showed there was no publication bias as demonstrated by asymmetrical funnel plot qualitatively (S2 Fig) and non-significant Egger’s regression test quantitatively (bias coefficient = -2.97, 95% CI: -15.06 to 9.13, p = 0.62). Two of the studies included had far-out values (47%) and outside values (30%) [Coefficient of Skewness = 1.81, p<0.001] **(S1 Fig).**

Regional variation showed significant effect on the estimated prevalence of *P*. *vivax* although there was high significant heterogeneity (*I*^2^ = 100%, p<0.0001) within each of the three main regions (Amhara, Oromia and SNNPR). SNNPR is a region where significantly highest (10%, 95%CI: 8.46–11.54%) pooled prevalence of *P*. *vivax* is documented (**S3 Fig**). Three studies (one of them contained a pair of studies) included in the review, which reported national/regional or more than one region prevalence were excluded from the locations/region’s analysis [58, 86, 87] (**S3 Fig**).

The different eco-epidemiological zones considered in the meta-analysis did appear to significantly affect the pooled estimate prevalence of *P*. *vivax* (*χ*^2^ = 18.65, df = 4, p = 0.0.01, *I*^2^ = 78.5%). Moreover, some studies reported from the highlands with occasional malaria epidemic zones (2000-2500m) contributed to the observed high prevalence of *P*. *vivax* (9.80%, 95%CI: 6.73–12.87%) compared to other eco-epidemiological zones (**S4 Fig**).

There were significant study setting differences (facility and community) among the studies (*χ*^2^ = 10.27, df = 1, p = 0.001, and *I*^*2*^ = 90.3%). Being diagnosed and treated at the health facility (health centers, health posts and hospitals) significantly (10.44%, 95%CI: 9.09–11.79%, p<0.0001) affected the overall pooled prevalence of *P*. *vivax*, although there was substantial unexplained high heterogeneity within the studies conducted at both settings (*I*^*2*^ = 100% for both). Hence, the validity of study setting effect estimate for each subgroup is uncertain as individual studies were inconsistent. However, a significant reduction in the prevalence of *P*. *vivax* (4.67%, 95%CI: 1.41–7.93%, p<0.0001) was observed in studies conducted at the population /community level (schools, and villages) (**S5 Fig**). Analysis of effects of study years on the pooled estimated prevalence of *P*. *vivax* revealed lack of statistically significant differences (p = 0.93, *I*^2^ = 0%) within the subgroups (**S6 Fig**).

### Meta-regression analysis

A meta-regression analysis was used to determine if sub-groups (geographical situation, altitudes of the study sites, years of the study and study settings) had an effect on the pooled prevalence of *P*. *vivax* in the country. Findings from this meta-regression analysis further confirmed the effect of the subgroups on the overall pooled *P*. *vivax* prevalence. Geographical situation of the studies (SNNPR region), study settings (study from health facilities compared to those from community), and studies reported from areas whose altitude ranges from 1500-1750m seemed to be associated with a significant increasing in the prevalence of *P*. *vivax* malaria in Ethiopia, but the remaining variables such as study year did not show significant effect on the pooled prevalence of *P*. *vivax*. Studies from altitude ranges from 2000 to 2500m showed comparatively higher prevalence of *P*, *vivax* next to altitude range from 1500–1750, although significant difference was not observed (Table 2).

**Table 2:** Meta-regression analysis of impact of subgroups on prevalence of *P*. *vivax* in Ethiopia, 2000–2020.

Subgroup	Covariate	Coefficient	SE	95% Lower CI	95% Upper CI	Z-value	P-value
	Intercept	8.05	1.45	5.21	10.895	5.55	0.00
Region	Oromia	0.65	1.09	-1.45	2.79	0.59	0.55
	SNNPR	2.60	1.02	0.6	4.61	2.54	0.01
Altitude	1500-1750m	3.30	1.44	0.49	6.11	2.3	0.02
	1750-2000m	-0.31	1.35	-2.95	2.33	-0.23	0.82
	2000-2500m	2.81	1.68	-0.5	6.11	1.67	0.09
	Mix	2.56	1.11	0.38	4.74	2.3	0.02
Study setting	Community	-5.94	1.004	-7.91	-3.97	-5.91	0.00
Study year	After 2010	-0.88	1.12	-3.07	1.31	-0.79	0.43

Note: CI = confidence interval, SE = standard error.

## Discussion

This study aimed to review the overall prevalence of *P*. *vivax* malaria infections in Ethiopia. For this purpose, any study that investigated the prevalence and epidemiology of malaria in the country, and which contained detailed data on *P*. *vivax* was included. The overall pooled prevalence of *P*. *vivax* malaria (mono-infection or mixed infection among symptomatic and asymptomatic patients) in Ethiopia was 8.93% (95% CI: 7.98–9.88%). Prevalence among *P*. *vivax* mono-infection alone was 7.98% (95% CI: 7.09–8.87%). These figures are much higher than the predicted endemicity values of *P*. *vivax* prevalence for Madagascar and Ethiopia, and parts of South Sudan and Somalia, which rarely exceed 2% [87]. Typically, the *P*. *vivax* parasite load in peripheral blood is very low as compared to *P*. *falciparum*, often hindering its diagnosis using conventional optic microscopy [88]. However, such low-level parasitemias are sufficient to act as reservoirs and sustain transmission of the parasite [89]. Although microscopy is still the gold standard tool for malaria diagnosis in Ethiopia, a more accurate approach for diagnosis would require the use of more sensitive techniques such as PCR or LAMP, capable of detecting submicroscopic carriage and mixed infections in areas where the two main parasites (*P*. *falciparum* and *P*. *vivax*) co-exist [90]. Given that most of the studies included in this review used microscopy as the chosen diagnostic tool, it is likely that the reported prevalence rates are an underestimate of the true prevalence of this parasite.

Ethiopia has variable topographic features that govern the distribution of malaria infection. Generally, it is agreed that malaria is endemic in areas with altitude lower than1500m (lowlands with seasonal/intense transmission) and rare in areas above 2000m (highland with occasional epidemic) [91]. However, in contrast to the general assumption, some studies reporting data from the highlands known for occasional malaria epidemics were found to contribute for a higher prevalence (9.80%, 95%CI: 6.73–12.87%) of *P*. *vivax*. This might be attributed to its survival ability in colder climate than other *Plasmodium* species [92]. A recent nationwide malaria epidemiological and interventional survey report confirms this finding, establishing the expansion of malaria to areas with altitude higher than 2000m [14], which were previously considered malaria free zones [93] and re-classified them as with moderate annual parasite incidence (APIs). The same report further indicated this as a new risk factor interfering with the current national malaria interventional activities [14]. A sero-prevalence study further strengthened the lack of significant differences in the transmission of *P*. *vivax* due to altitudinal variation (below or above 2000m) [93]. Rather, *P*. *vivax* showed direct relation with increasing elevation among children aged <5 years and high sero-positivity (20.9, 95% CI: 17.4–24.9) was observed at higher elevations [93]. The increasing evidence on the transmission of *P*. *vivax* in the areas traditionally considered as malaria free is an indication of the expansion of malaria transmission in Ethiopia to higher altitude settings. This expansion might be attributed to different developmental plans such as dam constructions, and the use of river water for irrigation purposes, deforestation, population pressures, and lack of appropriate environmental management system [86, 94], which could cause local environmental modifications contributing to the creation of new suitable vector breeding sites or expansion of mosquito’s habitat to non-endemic regions; besides changing human settlement pattern [95]. Malaria is one of the most climate sensitive diseases [96, 97] with significant associations between malaria incidence and temperature [96], relative humidity [97, 98] and rainfall [99], all of which do play a significant role in malaria transmission, which makes the vector controlling efforts very challenging. In addition, there are several Anopheles species with some different complexes, thus facilitating transmission into different ecological niches [100]. Furthermore, unlike other plasmodium species, *P*. *vivax* is capable of undergoing sporogonic development in the mosquito at lower temperatures [101] and able to expand to the highland areas. Growing evidence on *P*. *vivax* malaria distribution across other areas of Sub-Saharan Africa has further revealed that *P*. *vivax* appears to become proportionally more significant where overall malaria prevalence is lower [9].

Regional variation on *P*. *vivax* malaria prevalence was observed in the current review. In very recent years, significant reduction in *P*. *vivax* malaria burden has been predominantly observed in the Oromia region, as compared to the other regions [19, 72]. According to the National Strategic Plan for Malaria Prevention, Control and Elimination in Ethiopia, the malaria burden was significantly reduced over three survey years (2007, 2011 and 2015) with 0.3% nationwide prevalence in the year 2015 [90]. This figure is relatively lower than reports made from other regions including SNNPR (0.5%), Amhara (0.8%), Benshangul (2.7%) and Gambella (6%) in the same year [90].

Compared to the national report, the prevalence of *P*. *vivax* malaria infection reported in the current review is much higher. This is due to the fact that the national report was the overall national malaria prevalence, which included only recent data (after malarial morbidity and mortality burden started decreasing) from all malaria transmission settings (low, middle, and high). But, this review only focused on prevalence of *P*. *vivax* malaria infection and included almost all studies conducted at high malaria transmission areas, and the prevalence data of 20 years. The recent national sero-prevalence analysis by region supports this finding, with lower *P*. *vivax* sero-prevalence documented in Oromia than in Amhara (36.7% (95% CI: 30.0–44.1) and SNNPR regions [92], although the detected antibodies might not correspond adequately to the existing infection prevalence.

Following the rise in malaria prevalence as observed in the year 2010/11, the deployment of malaria interventions already initiated in Ethiopia was boosted. This included the distribution of free ITN, IRS, and RDTs as a supplement for malaria diagnosis in remote areas, and the scale-up of ACT deployment and training of health extension workers [102]. As a result, the overall national malaria burden decreased from 0.5% prevalence in 2011 to 0.3% in 2015 [90]. Our meta-analysis on studies whose survey years were before and after the scaling-up of national malaria intervention activities did not show significant effect on the pooled estimated prevalence of *P*. *vivax* in Ethiopia. However, results from meta-regression indicated that prevalence of *P*. *vivax* observed after the scaling up of the interventional activities in Ethiopia, showed significant reduction. This finding is in agreement with the global *P*. *vivax* malaria burden reduction observed (41.6% reduction from 2000 to 2017) in most endemic areas [103]. Although the trend showed a declining pattern, burden due to *P*. *vivax* in Ethiopia appears considerable, and will cause enormous challenges, calling for careful regular surveillance by concerned bodies. Mainly it’s apparent complex parasite biology, pathophysiology, treatment response, the raising problem of Duffy negative individuals that are now infected by *P*. *vivax* and transmission patterns [104] will make its future eradication goal very challenging. In addition, the hypnozoite‘s dormant liver stages, responsible for the potential repeated relapses that can occur within weeks, months, or many years after the initial inoculation, blur our current understanding of *P*. *vivax* epidemiology, and will not be affected unless specific radical cure is conducted [102]. In the absence of such anti-hypnozoite drugs, the current first line drugs used in Ethiopia for *P*.*vivax* malaria, be it chloroquine or other artemisinin based-combination therapies, will not affect the liver stage hypnozoites [9], thus hindering its adequate control. In addition, ITN and IRSs currently in use might not be efficient in completely preventing new infection, in general, and the relapse from liver stages in particular [9], mosquito species that transmit *P*. *vivax* bite mostly outdoors and which also changed its biting time from midnight to dawn [105]. Some populations of *An*. *arabiensis* were reported to even avoid fatal insecticide exposure [106, 107].

### Strengths and limitations of the study

To the best of our knowledge, this is the first detailed systematic review and meta-analysis of only *P*. *vivax* epidemiology in Ethiopia that included facility and community level studies. A recent systematic review and meta-analysis by Deresse and Girma, [108] assessed (using 35 studies) the prevalence of *P*. *falciparum* and *P*. *vivax* in Ethiopia and found 25.8% prevalence all together. Its main objective was to show a general picture on malaria prevalence in Ethiopia. Hence *P*. *vivax* prevalence/epidemiology was not uniquely reviewed, analyzed or presented separately in the study. Furthermore, the study didn’t include the major databases such as Web of Science, Scopus, and EMBASE, but only retrieved articles from PubMed and Google scholar. In addition, it did not assess the role of subgroups such as location, eco-epidemiological zones, study setting and survey years, on the overall pooled prevalence of malaria, in general, and *P*. *vivax* in particular. The omission of subgroups appears to have significant impact, given that these subgroups showed a significant role on the estimated prevalence of *P*. *vivax* in our analysis. Hence, the strength of this review is the fact that it included many other new studies to date (n = 44) on *P*. *vivax* in Ethiopia besides the 35 studies included in the previous review and portrayed the epidemiological distribution of *P*. *vivax* nationwide [108]

The major limitation of this review was that about one third of the included studies depended on data extracted from retrospective medical case records, reviewed to investigate the prevalence and trends of malaria. Although case record reviews are the most universally used method for prevalence studies, it is often challenging to obtain, in a standardized way, all required data about the individual patient, including socio-demographic and clinical data, how target groups were identified, recruited and the exact diagnostic tools used at the time of enrollment of each participant. In addition, for some of the studies included in the review, their main objective was not set to assess the prevalence or geographical distribution or epidemiological trends of malaria. Some were designed to show association between malaria prevalence and ABO blood groups/helminthic infection/HIV infection/ ITN utilization /hematological profile of malaria patients/ drug efficacy evaluation against *P*. *vivax*/or comparative evaluation of different malaria diagnostic tests or tools (microscopy Vs PCR). Data from this kind of studies often don’t allow an adequate evaluation of the quality criteria set for prevalence/observational studies. Thus, they were included in the review only if they contained data on prevalence of malaria and different *Plasmodium* species. Moreover, significant heterogeneity of the eligible studies observed in this review may require further analysis. Finally, the exclusion of unpublished studies as well as interventional studies may lead, potentially, to loss of substantial data.

## Conclusions

The overall estimated prevalence of *P*. *vivax* was 8.93% (95%CI: 7.98–9.88). Most of the studies included in the current review met the quality criteria and there was no publication bias. This parasite has historically been widely distributed in the central west region of Ethiopia, and is now steadily extending to the North West and South West regions of the country. Oromia, Amhara and SNNPR are the three major regions where *P*. *vivax* has spread predominantly with wide-ranging prevalence. *P*. *vivax* epidemiology has shown the trend of expansion to the highland, causing occasional malaria epidemics, although the existing deployed interventions seem to have an impact on prevalence of this parasite.

## Supporting information

S1 Table Summary of search keywords/terms. (DOCX)

S2 Table Excluded studies and reasons for exclusion of studies on prevalence of *P*. *vivax* infection in Ethiopia. (DOCX)

S3 Table Risk bias assessment based on the Prevalence Critical Appraisal Instrument of studies on prevalence of *P*. *vivax* infection in Ethiopia. (DOCX)

S1 Fig Boxplot of studies on prevalence of *P*. *vivax* infection in Ethiopia. (DOCX)

S2 Fig Funnel plot for publication bias assessment of studies on prevalence of *P*. *vivax* infection in Ethiopia. (DOCX)

S3 Fig Pooled estimates of prevalence of *P*. *vivax* for different locations/regions of Ethiopia. (DOCX)

S4 Fig Estimate prevalence of P. vivax in different eco-epidemiological zones of Ethiopia. (DOCX)

S5 Fig Prevalence of *P*. *vivax* at different study settings in Ethiopia. (DOCX)

S6 Fig Prevalence of *P*. *vivax* with respect to year of survey in Ethiopia. (DOCX)
