# Supplementary material for: Plasmodium vivax epidemiology in Ethiopia 2000-2020: A systematic review and meta-analysis
Source: PLoS Negl Trop Dis. 2021 Sep 15;15(9):e0009781. doi: 10.1371/journal.pntd.0009781 (PMC8476039; doi:10.1371/journal.pntd.0009781)
Supplement: S1 Table — (DOCX) [file pntd.0009781.s001.docx]

**Supplementary Table 1: Summary of search keywords/terms**

| **Database** | **Search strategy** |
| --- | --- |
| PubMed= 1,021 | (Plasmodium[Title/Abstract] OR malaria*[Title/Abstract] OR "Malaria"[MeSH Terms] OR "Malaria, Vivax"[MeSH Terms]) AND (Ethiopia*[Title/Abstract] OR Ethiopia*[Affiliation] OR "Ethiopia"[MeSH Terms]) AND ("2000/01/01"[Date – publication]: "2020/12/31"[Date – publication]) |
| Embase = 1,250 | (Plasmodium:ti,ab,kw OR malaria*:ti,ab,kw OR 'malaria'/exp) AND (Ethiopia*:ti,ab,kw,ca OR 'ethiopia'/exp) AND [2000-2020]/py |
| Web of Science (Core Collection) = 1,356  Citation Indexes: SCI-EXPANDED, SSCI, A&HCI, CPCI-S, CPCI-SSH, BKCI-S, BKCI-SSH, ESCI, CCR-EXPANDED, IC. | TS=(Plasmodium OR malaria*) AND (TS=Ethiopia* OR CU=Ethiopia) AND PY=2000-2020 |
| Scopus = 1,298 | TITLE-ABS-KEY(Plasmodium OR malaria*) AND (TITLE-ABS-KEY(Ethiopia*) OR AFFILCOUNTRY(Ethiopia)) AND PUBYEAR AFT 1999 AND PUBYEAR BEF 2021 |
| African Journals Online (AJOL) = 7 | Manually searched |
