# Supplementary material for: Plasmodium vivax epidemiology in Ethiopia 2000-2020: A systematic review and meta-analysis
Source: PLoS Negl Trop Dis. 2021 Sep 15;15(9):e0009781. doi: 10.1371/journal.pntd.0009781 (PMC8476039; doi:10.1371/journal.pntd.0009781)
Supplement: S2 Table — (DOCX) [file pntd.0009781.s002.docx]

**S2_Table. Excluded studies and reasons for exclusion of studies on prevalence of *P. vivax* infection in Ethiopia**

| **S/N** | **Authors** | **Title** | **Year** | **Journal** | **Reason for exclusion** |
| --- | --- | --- | --- | --- | --- |
| 1 | Addissie et al., | Malaria and HIV co-infection in Hadya Zone, southern Ethiopia | 2007 | Ethiopian Medical Journal | Lack separate prevalence data for P. vivax |
| 2 | Alemu et al | Malaria infection has spatial, temporal, and spatiotemporal heterogeneity in unstable malaria transmission areas in northwest Ethiopia. | 2013 | PLoS One | Lack separate prevalence data for P. vivax |
| 3 | Alemu et al., | Malaria helminth co-infections and their contribution for aneamia in febrile patients attending Azzezo health center, Gondar, Northwest Ethiopia: a cross sectional study. | 2012 | Asian Pac J Trop Med. | No separate data for each plasmodium species |
| 4 | Argaw et al. | Management of uncomplicated malaria in private health facilities in North-West Ethiopia: a clinical audit of current practices. , 2019, | 2019 | BMC Heal Service Res | Denominator is not clear. |
| 5 | Ashton et al | Geostatistical modeling of malaria endemicity using serological indicators of exposure collected through school surveys. | 2015 | Am J Trop Med Hyg. | Malaria prevalence data is a duplicate/the same as the study from Ashton et al . 2011 |
| 6 | Asmamaw et al. | Prevalence of malaria and HIV among pregnant women attending antenatal clinics at felege hiwot referral hospital and addis zemen health center in northwest of Ethiopia | 2013 | International Journal of Life Sciences Biotechnology and Pharma Research | There were no P. vivax infected pregnant women |
| 7 | Assefa et a. | Assessment of subpatent Plasmodium infection in northwestern Ethiopia | 2020 | Malaria Journal | Lack separate prevalence data for P. vivax |
| 8 | Ayele | Malaria Epidemics in Dembia, Northwest Ethiopia 1952 – 1953 | 2017 | Ethiopian Journal of Health Development | Reported old data (1952-1953) |
| 9 | Ayele et al., | Prevalence and risk factors of malaria in Ethiopia | 2012 | Malaria Journal | Lack of malaria prevalence data |
| 10 | Ayele et al., | Prevalence and risk factors of malaria in Ethiopia | 2010 | Malaria Journal | Lack of malaria prevalence data |
| 11 | Bansil et al. | Malaria case investigation with reactive focal testing and treatment: Operational feasibility and lessons learned from low and moderate transmission areas in Amhara Region, Ethiopia | 2018 | Malaria Journal | Lack of malaria prevalence data |
| 12 | Barreiro et al. | Malaria and severe anemia over eight years at Gambo Rural Hospital, southern Ethiopia | 2017 | Pathogens and Global Health | Lack separate prevalence data for P. vivax |
| 13 | Birhanu et al | Quantifying malaria endemicity in Ethiopia through combined application of classical methods and enzyme-linked immunosorbent assay: an initial step for countries with low transmission initiating elimination program | 2016 | Malar J | Lack prevalence data for each plasmodium species |
| 14 | Degarege et al | Malaria severity status in patients with soil-transmitted helminth infections. | 2009 | Acta Tropica | There is a denominator of 1802 febrile patients, of whom 502 had malaria. We only have the species information of 458 subjects |
| 15 | Degarege et al. | Malaria and helminth co-infection and nutritional status of febrile patients in Southern Ethiopia | 2014 | Journal of Infection and Public Health, | Lack separate prevalence data for P. vivax |
| 16 | Degarege et al. | Malaria and helminth co-infections in outpatients of Alaba Kulito Health Center, southern Ethiopia: a cross sectional study | 2010 | [BMC Res Notes](https://www.ncbi.nlm.nih.gov/pmc/articles/PMC2902494/) | There is a denominator of 1802 febrile patients, of whom 502 had malaria. We only have the species information of 458 subjects |
| 17 | Degefa et al | Malaria incidence and assessment of entomological indices among resettled communities in Ethiopia: a longitudinal study. | 2015 | Malaria Journal | Lack separate prevalence data for P. vivax |
| 18 | Demissie et al | [Assessment of public health implication of malaria-geohelminth co-infection with an emphasis- on hookworm-malaria anemia among suspected malaria patients in asendabo, southwest Ethiopia.](https://reference.medscape.com/viewpublication/5441) | 2009 | Ethiop Med J. | No access to full article |
| 19 | Deribew et al. | Malaria and under-nutrition: a community-based study among under-five children at risk of malaria, south-west Ethiopia. | 2010 | PLoS One | Lack of malaria prevalence data |
| 20 | File et al. | A retrospective analysis on the transmission of Plasmodium falciparum and Plasmodium vivax: The case of Adama City, East Shoa Zone, Oromia, Ethiopia | 2019 | Malaria Journal | Lack separate prevalence data for P. vivax |
| 21 | Gari et al., | Malaria increased the risk of stunting and wasting among young children in Ethiopia: Results of a cohort study. | 2018 | PLoS One | Lack of malaria prevalence data/it contained incidence data |
| 22 | Getachew et al | Prevalence of soil transmitted helminthiasis and malaria co-infection among pregnant women and risk factors in Gilgel Gibe Dam area, southwest Ethiopia. | 2013 | BMC Res Notes. | No separate prevalence data for each plasmodium species |
| 23 | Getaneh et al. | Malaria Parasitemia in Febrile Patients Mono- and Coinfected with Soil-Transmitted Helminthiasis Attending Sanja Hospital, Northwest Ethiopia | 2020 | J Parasitol Res. | Lack of malaria prevalence data |
| 24 | [Girma et al.,](javascript:;) | Prevalence and Epidemiological Characteristics of Asymptomatic Malaria Based on Ultrasensitive Diagnostics: A Cross-sectional Study | 2019 | Clinical Infectious Diseases, | Lack of malaria prevalence data |
| 25 | Haile et al | Population Movement as a Risk Factor for Malaria Infection in High-Altitude Villages of Tahtay-Maychew District, Tigray, Northern Ethiopia: A Case-Control Study. | 2017 | Am J Trop Med Hyg. | No separate prevalence data for each plasmodium species |
| 26 | Kassa et al. | Characterization of peripheral blood lymphocyte subsets in patients with acute Plasmodium falciparum and P. vivax malaria infections at Wonji Sugar Estate, Ethiopia. | 2006 | Clin Vaccine Immunol. | Lack separate prevalence data for P. vivax |
| 27 | Keffale et al. | Serological evidence for a decline in malaria transmission following major scale-up of control efforts in a setting selected for Plasmodium vivax and Plasmodium falciparum malaria elimination in Babile district, Oromia, Ethiopia | 2019 | Transactions of the Royal Society of Tropical Medicine and Hygiene | Lack separate prevalence data for P. vivax |
| 28 | Khogali et al | Detection of malaria in relation to fever and grade of malnutrition among malnourished children in Ethiopia. | 2011 | Public Health Action. | No separate prevalence data for each plasmodium species |
| 29 | Kibret et al. | The impact of a small-scale irrigation scheme on malaria transmission in Ziway area, Central Ethiopia | 2010 | Tropical Medicine and International Health | Lack separate prevalence data for *P. vivax* |
| 30 | Lo et al. | Transmission dynamics of co-endemic Plasmodium vivax and P. falciparum in Ethiopia and prevalence of antimalarial resistant genotypes | 2017 | PLoS Neglected Tropical Diseases | Lack separate prevalence data for *P. vivax* |
| 31 | Loha et al | Long-lasting insecticidal nets and indoor residual spraying may not be sufficient to eliminate malaria in a low malaria incidence area: results from a cluster randomized controlled trial in Ethiopia | 2019 | Malaria Journal | Malaria prevalence data is the same with Solomon et al., 2019 |
| 32 | Lyon et al. | Temperature suitability for malaria climbing the Ethiopian Highlands | 2017 | Environmental Research Letters | Lack of malaria prevalence data |
| 33 | [Mavrogordato et al.](https://pubmed.ncbi.nlm.nih.gov/?term=Mavrogordato+A&cauthor_id=22609735) | A cluster of Plasmodium vivax malaria in an expedition group to Ethiopia: prophylactic efficacy of atovaquone/proguanil on liver stages of P. vivax | 2012 | J Infect | Lack of malaria prevalence data |
| 34 | Moha et al | Assessment of malaria hazard, vulnerability, and risks in Dire Dawa City Administration of eastern Ethiopia using GIS and remote sensing. | 2020 | Appl Geomat | Lack of malaria prevalence data |
| 35 | Mulu et al | Epidemiological and clinical correlates of malaria-helminth co-infections in Southern Ethiopia. | 2013 | Malaria Journal | There is no data for the sampled/total population diagnosed for malaria |
| 36 | Nega et al | Anemia associated with asymptomatic malaria among pregnant women in the rural surroundings of Arba Minch Town, South Ethiopia. | 2015b | BMC Res Notes. | Malaria prevalence data is a duplicate of Nega et al., 2015a |
| 37 | Shargie et al | Malaria prevalence and mosquito net coverage in Oromia and SNNPR regions of Ethiopia. | 2008 | BMC Public Health | Lack of malaria prevalence data |
| 38 | Sleshi et al. | Malaria microscopy performance in self-presenting febrile patients at four health facilities in Fentale district of East Shewa, Ethiopia | 2012 | Ethiopian Medical Journal | Lack separate prevalence data for *P. vivax* |
| 39 | Solomon et al. | Spatiotemporal clustering of malaria in southern-central Ethiopia: A community-based cohort study. | 2019 | PLoS One. | There is no point Prevalence data |
| 40 | Tadesse et al | The Relative Contribution of Symptomatic and Asymptomatic Plasmodium vivax and Plasmodium falciparum Infections to the Infectious Reservoir in a Low-Endemic Setting in Ethiopia. | 2018 | Clin Infect Dis. | No separate prevalence data for each plasmodium species |
| 41 | Tadesse et al. | Submicroscopic carriage of Plasmodium falciparum and Plasmodium vivax in a low endemic area in Ethiopia where no parasitaemia was detected by microscopy or rapid diagnostic test | 2015 | Malaria Journal | Lack of malaria prevalence data |
| 42 | Tadesse et al. | The shape of the iceberg: Quantification of submicroscopic Plasmodium falciparum and Plasmodium vivax parasitaemia and gametocytaemia in five low endemic settings in Ethiopia | 2017 | Malaria Journal | Lack separate prevalence data for *P. vivax* |
| 43 | Taffese et al. | Malaria epidemiology and interventions in Ethiopia from 2001 to 2016 | 2018 | Infectious Diseases of Poverty | National report, aggregate data, which lack separate information for each plasmodium species |
| 44 | Tajebe et al. | Detection of mixed infection level of Plasmodium falciparum and Plasmodium vivax by SYBR Green I-based real-Time PCR in North Gondar, north-west Ethiopia | 2014 | Malaria Journal | Lack separate prevalence data for *P. vivax* |
| 45 | Tefera et al | Economic Burden of Malaria and Associated Factors Among Rural Households in Chewaka District, Western Ethiopia | 2020 | Clinicoecon Outcomes Res. | Lack of malaria prevalence data |
| 45 | Tekeste and Petros | The ABO blood group and Plasmodium falciparum malaria in Awash, Metehara and Ziway areas, Ethiopia | 2010 | Malaria journal | There is no differentiated prevalence data |
| 47 | Tesfay et al | Trend analysis of malaria prevalence in Raya Azebo district, Northern Ethiopia: a retrospective study. | 2018 | BMC Res Notes | There is no data for the sampled/total population diagnosed for malaria |
| 48 | Tesfaye et al. | Common mental disorder symptoms among patients with malaria attending primary care in Ethiopia: A cross-sectional survey | 2014 | PLoS ONE | Lack of malaria prevalence data |
| 49 | Tilahun et al | Prevalence of asymptomatic Plasmodium species infection and associated factors among pregnant women attending antenatal care at Fendeka town health facilities, Jawi District, North west Ethiopia: A cross-sectional study. | 2020 | PLoS One. | No separate prevalence data for each plasmodium species |
| 50 | Tilaye and Deressa | Prevalence of urban malaria and associated factors in Gondar Town, Northwest | 2007 | Ethiopian Medical Journal. | No access to full article |
| 51 | Toyama et al. | Sharp decline of malaria cases in the Burie Zuria, Dembia, and Mecha districts, Amhara Region, Ethiopia, 2012-2014: descriptive analysis of surveillance data | 2016 | Malaria Journal | Lack of malaria prevalence data |
| 52 | Wale and Mindaye | Impact of insecticide-treated bednet use on malaria prevalence in Benishangul-Gumuz regional state, Ethiopia. | 2016 | J Vector Borne Dis. | No denominator (sample size is unknown) |
| 53 | Yalew et al., | Current and cumulative malaria infections in a setting embarking on elimination: Amhara | 2017 | Malaria Journal | There is no data for the sampled/total population diagnosed for malaria |
| 54 | Yukich et al. | Travel history and malaria infection risk in a low-transmission setting in Ethiopia: a case control study. | 2013 | Malar J, | Denominator for malaria positive cases not known |
