# Supplementary material for: Plasmodium vivax epidemiology in Ethiopia 2000-2020: A systematic review and meta-analysis
Source: PLoS Negl Trop Dis. 2021 Sep 15;15(9):e0009781. doi: 10.1371/journal.pntd.0009781 (PMC8476039; doi:10.1371/journal.pntd.0009781)
Supplement: S3 Table — (DOCX) [file pntd.0009781.s003.docx]

**S3_Table. Risk bias assessment based on the Prevalence Critical Appraisal Instrument of studies on prevalence of *P. vivax* infection in Ethiopia**

| Author and year | Q1) Target population identification | Q2) Recruitment adequacy | Q3) Sample size adequacy | Q4) Subject & setting description | Q5) Coverage of the identified sample | Q6) Standard condition measurement | Q7) Reliable condition measurement | Q8) Adequate statistical analysis | Q9) Confounders accounted for | Q10) Sub-groups identified | Quality items met (n/10) | Risk level | Remark |
| --- | --- | --- | --- | --- | --- | --- | --- | --- | --- | --- | --- | --- | --- |
| Abossie et al., 2019 | N | Y | Y | Y | N | Y | Y | N | Y | Y | 7 | Medium | Target population is not clear, <5 yrs are from 0-5, not from 12 months to 5yrs, proper sample size calculation and systematic sampling technique, in adequate statistical analysis, |
| Addisu et al., 2020 | NA | NA | Y | N | N | Y | N | N | NA | Y | 3 | High | Retrospective study from recorded data, very brief report. Detailed procedure is missing, in adequate data analysis, |
| Alelign et al., 2018 | NA | NA | Y | Y | Y | Y | Y | Y | Y | Y | 8 | Low | Data obtained from recorded medical cards (secondary data), Very brief report. Detailed procedure is not described, in adequate data analysis, microscopy, a standard diagnostic tool was used, experts involve in data collection |
| Alemayehu et al., 2015 | Y | UC | Y | Y | Y | Y | Y | Y | UC | Y | 8 | Low | Confounding factors associated to co-infection are not well addressed, procedure for patient’s enrollment was not described in detail |
| Alemu & Mama, 2018 | Y | Y | Y | N | Y | Y | N | N | Y | UC | 6 | Medium | Study setting and malaria burden not described well |
| Alemu et al., 2011 | Y | Y | Y | Y | Y | Y | Y | Y | Y | N | 9 | Low | Good, but study subjects are not well described |
| Alemu et al., 2012 | Y | Y | Y | Y | Y | Y | Y | N | N | Y | 8 | Low | Potential confounding factors were not adequately assessed. Detailed statistical analysis is missing |
| Beyene et al., 2018 | NA | NA | Y | Y | Y | Y | Y | N | Y | Y | 7 | Medium | Retrospective study from recorded data, with very brief statistical analysis tools employed |
| Alemu et al., 2014 | Y | Y | Y | Y | Y | Y | Y | Y | Y | Y | 10 | Low | Complete |
| Alkadir et al., 2020 | NA | NA | Y | Y | Y | NA | Y | Y | Y | Y | 7 | Medium | A retrospective study, its data from medical records and difficult to assess some quality criteria |
| Animut et al., 2009 | N | N | Y | N | N | Y | Y | N | NA | Y | 4 | High | All age groups were the target but, only included 3-17yrs, no description of study area, no sample size calculated, no sampling tech., inadequate data analysis |
| Argaw et al. 2016 | UC | UC | UC | Y | Y | Y | Y | Y | Y | Y | 7 | Medium | Target groups, sample size calculation and sampling technique are not clearly indicated. |
| Aschale et al., 2018 | Y | Y | Y | Y | Y | Y | Y | Y | Y | Y | 10 | Low | All parts are complete |
| Aschale et al., 2019 | Y | Y | Y | Y | Y | Y | Y | N | Y | Y | 9 | Low | Inadequate statistical analysis |
| Ashton et al. 2011 | Y | Y | Y | Y | Y | Y | Y | Y | Y | Y | 10 | Low | All parts are complete |
| Assefa et al., 2015 | Y | UC | UC | Y | Y | Y | Y | Y | Y | UC | 7 | Medium | Procedure/method mainly adopted from other reference (WHO) and detailed information were missing |
| Awoke & Arota, 2019 | Y | N | Y | N | Y | Y | N | N | N | UC | 4 | High | Data analysis tools were not exhaustive and appropriate tools such as logistic regression was not used, detail description of study setting and population are missing, sampling technique is not clear. |
| Ayalew et al., 2016 | Y | Y | Y | Y | Y | N | N | Y | Y | Y | 8 | Low | Instrument used for measurement is not standard and reliable. May not show the current malaria infection |
| Belete and Roro., 2016 | Y | Y | Y | Y | Y | Y | Y | Y | Y | N | 9 | Low | Subpopulation were not identified and considered |
| Berhanie et al., 2014 | Y | N | N | Y | N | Y | Y | Y | Y | Y | 7 | Medium | Sample size were not calculated, small and not enough to draw conclusion out of it, its coverage is limited |
| Dabaro et al., 2020 | NA | NA | Y | Y | Y | NA | Y | Y | N | Y | 6 | Medium | Retrospective study from recorded data, confounders such as interventional activities accountable for the malaria burden reduction are missing, |
| Debo & Kassa, 2016 | Y | Y | Y | Y | Y | Y | Y | Y | Y | UC | 9 | Low | Fulfills most of the criteria |
| Degarege et al., 2011 | Y | N | N | Y | Y | Y | N | N | N | Y | 5 | Medium | Sample size is not properly calculated, no clear procedure for sampling techniques, in adequate statistical analysis, and confounding factors are not identified |
| Degarege et al., 2012 | Y | N | Y | Y | Y | Y | Y | Y | N | N | 7 | Medium | Confounding factors such as nutritional status … were not adequately assessed, Sampling technique is not mentioned, all data were not analyzed |
| Delil et al., 2016 | Y | Y | Y | Y | Y | Y | Y | Y | Y | Y | 10 | Low | Complete |
| Demissie & Ketema, 2016 | Y | N | Y | Y | Y | Y | N | Y | N | N | 6 | Medium | No sampling technique, statistical tool used was relative risk, other tests such as logistic regression are more reliable and valid |
| Derbie & Alemu, 2017 | NA | NA | Y | N | Y | NA | Y | Y | Y | Y | 6 | Medium | brief retrospective study from recorded data, description about disease in the study area is missing |
| Legesse et al., 2015 | NA | NA | Y | Y | Y | NA | Y | N | Y | Y | 6 | Medium | A retrospective study, its data from medical records and difficult to assess some quality criteria. Lack adequate statistical analysis |
| Dufera et al., 2020 | Y | Y | Y | Y | Y | Y | Y | Y | Y | Y | 10 | Low | Complete |
| Ergete et al., 2018 | NA | NA | Y | N | Y | NA | Y | Y | N | UC | 4 | High | A brief retrospective study from recorded data, description about disease in the study area is missing, confounders were not considered |
| Esayas et al., 2020a | Y | Y | Y | Y | Y | Y | N | N | Y | Y | 8 | Low | Detailed data collection, but reliable and valid data analysis tools are missing |
| Esayas et al., 2020b | NA | NA | Y | Y | Y | NA | Y | Y | Y | Y | 7 | Medium | A retrospective study, its data from medical records and difficult to assess some quality criteria |
| Feleke et al., 2018 | NA | NA | Y | Y | Y | NA | Y | Y | N | N | 5 | Medium | 5 years trend analysis, use secondary data from recorded medical record. Potential confounding factors such as effect of interventional activities were not assessed. |
| Feleke et al., 2020 | Y | Y | Y | Y | Y | Y | UC | Y | Y | UC | 9 | Low | Subpopulation identification are not clear |
| Ferede et al., 2013 | NA | NA | Y | Y | N | NA | N | N | Y | UC | 3 | High | It is brief retrospective study on recorded data from health facility. Potential confounding factors such as effect of interventional activities were not assessed. |
| Gebretsadik et al., 2018 | NA | NA | Y | Y | N | NA | N | N | N | N | 2 | High | 8 years trend analysis, use secondary data from recorded medical record, potential confounding factors were not assessed, a single statistical tool was not used for data analysis |
| Geleta & Ketema, 2016 | Y | N | N | Y | Y | Y | Y | Y | Y | UC | 7 | Medium | No sample size determination and sampling technique. Detailed and appropriate data analysis, but unclear about the identification of subpopulation |
| Golesa & White, 2017 | Y | N | Y | Y | Y | Y | Y | Y | Y | Y | 9 | Low | Method for sampling technique was not indicated |
| Gonitie et al., 2020 | Y | Y | Y | Y | Y | N | N | Y | Y | Y | 8 | Low | Method used for diagnosis is not standard and reliable, don’t tell the actual/current infection |
| Haile et al., 2020 | NA | NA | Y | Y | Y | NA | Y | Y | N | Y | 6 | Medium | All confounding factors such as seasonal variation, were not exhaustively assessed |
| Haji et al., 2016 | Y | N | Y | Y | Y | Y | Y | Y | Y | UC | 8 | Low | Method for sampling technique was not indicated, identification of subpopulation is not clear |
| Hassen & Dinka, 2020 | NA | NA | Y | Y | Y | NA | N | N | N | Y | 4 | High | retrospective study from recorded data, data were included in the analysis, but reliable, valid and appropriate data analysis tools were missing |
| Hawaria et al., 2018 | NA | NA | Y | Y | Y | NA | Y | Y | Y | Y | 7 | Medium | retrospective study from recorded data |
| Ifa, 2018 | NA | NA | Y | N | Y | NA | N | N | N | N | 2 | High | Very brief retrospective study from recorded data, description about disease in the study area is missing, reliable and valid data analysis tools are missing, confounders and subpopulations such as d/t age groups, gender and interventional activities were not identified. |
| Jemal & Ketema, 2019 | NA | NA | Y | Y | Y | NA | N | N | N | Y | 4 | High | All data were included for analysis, but the data analysis tools were not exhaustive, confounders were not identified and accounted |
| Kalil et al., 2020 | NA | NA | Y | Y | Y | NA | Y | N | Y | Y | 6 | Medium | A retrospective study, its data from medical records, appropriate data analysis tools to assess association among variables such as logistic regression is missing |
| Kamrunamoorthi & Bekele, 2009 | Y | N | Y | Y | Y | Y | N | N | N | UC | 4 | High | Sampling technique not clear, valid, reliable and appropriate data analysis tools were not exhaustively used |
| Lankir et al., 2020 | NA | NA | Y | Y | NA | NA | NA | N | Y | Y | 4 | High | A retrospective study from recorded data and most of quality criteria were difficult to measure |
| Lo et al. 2015 | Y | N | Y | N | Y | Y | Y | Y | Y | UC | 7 | Medium | Sampling technique is not clear, description of study setting and study population were missing |
| Mekonnen et al., 2014 | Y | N | Y | Y | Y | Y | Y | Y | N | UC | 7 | Medium | Sampling technique is not clear, confounding factors were not identified |
| Minwuyelet et al., 2020 | Y | Y | Y | Y | Y | Y | Y | Y | N | N | 8 | Low | Potential confounding factors and subgroups were not exhaustively addressed. |
| Nega et al., 2015 | Y | Y | Y | Y | Y | Y | Y | Y | Y | Y | 10 | Low | complete |
| Schicker et al., 2015 | Y | Y | Y | Y | Y | N | N | Y | Y | Y | 8 | Low | Method used for diagnosis is not standard and reliable, don’t tell the actual/current infection |
| Shamebo & Petros | NA | NA | Y | Y | NA | NA | N | N | N | N | 2 | High | A Very brief retrospective study from medical records, data were not properly extracted, valid, reliable and appropriate data analysis were missing, potential confounders were not identified |
| Shiferaw et al., 2018 | NA | NA | Y | Y | NA | NA | NA | N | N | Y | 3 | High | A retrospective study from recorded data and most of quality criteria were difficult to measure. In addition, lack adequate statistical analysis |
| Solomon et al., 2019 | Y | Y | Y | Y | Y | Y | Y | Y | Y | Y | 10 | Low | complete |
| Solomon et al., 2020 | Y | UC | Y | Y | Y | Y | Y | Y | Y | Y | 9 | Low | Sampling technique was unclear |
| Tadesse and Tadesse, 2013 | Y | N | N | Y | N | Y | Y | Y | UC | UC | 5 | Medium | No sample size determination, no sampling technique, association between variables need other statistical tools eg. Regression, correlation… |
| Tadesse et al., 2015 | Y | N | N | Y | Y | Y | Y | Y | UC | UC | 6 | Medium | No formal sample size calculation was performed and unclear sampling technique, identification of confounding factors and subpopulation were not clear |
| Tadesse et al., 2017 | Y | Y | Y | Y | Y | Y | Y | Y | Y | Y | 10 | Low | Complete |
| Tesfaye et al., 2011 | Y | Y | Y | Y | N | Y | N | N | N | Y | 6 | Medium | No reliable, valid and appropriate data analysis tools were used, potential confounding factors were not assessed well. |
| Tesfa et al., 2018 | NA | NA | Y | Y | NA | NA | NA | Y | Y | Y | 5 | Medium | As this is retrospective study from recorded data, it is difficult to measure some of the quality parameters |
| Tesfaye et al., 2019 | Y | N | Y | N | N | Y | N | N | N | Y | 4 | High | Study setting and population were not well described, sampling technique was unclear, no valid and reliable data analysis tool, confounding factors were not identified, |
| Tuasha et al., 2019 | Y | N | Y | Y | Y | Y | Y | Y | N | Y | 8 | Low | Sampling technique is not clear, confounding factors related to personal hygiene were not exhaustively identified, |
| Woday et al., 2019 | N | Y | Y | Y | Y | Y | Y | Y | Y | Y | 9 | Low | According to topic of the study the target groups are all children, not only children <5 years, |
| Wondimeneh et al., 2018 | Y | N | Y | Y | Y | Y | Y | Y | Y | UC | 9 | Low | Subpopulation identification not clear, convenient sampling method used |
| Woyessa et al., 2012 | Y | Y | Y | Y | Y | Y | Y | Y | N | N | 8 | Low | Potential confounding factors and subgroups were not identified and accounted. |
| Yehualaw et al., 2009 | N | Y | Y | Y | Y | Y | Y | Y | Y | Y | 9 | Low | Target group are children, but topic doesn’t show that |
| Yimer et al., 2015 | Y | Y | Y | Y | Y | Y | N | N | Y | Y | 8 | Low | Data analysis tools were not exhaustive, predictor variables were not identified |
| Yimer et al., 2017 | NA | NA | Y | N | N | NA | Y | Y | N | Y | 4 | High | Disease nature and population are not well described, inadequate data analysis and data presentation, no trained data collector or analyzer |
| Zerihun et al., 2011 | Y | N | N | Y | Y | Y | N | N | N | Y | 5 | Medium | There is no sample size calculation and sampling technique, |
| Zhou et al., 2016 | Y | Y | Y | Y | Y | Y | Y | Y | Y | Y | 10 | Low | Complete |

Note: Y = Fulfills the criteria, N= No fulfills the criteria, UC= Unclear, NA= Not applicable,
