# Supplementary figures and images for: Plasmodium vivax epidemiology in Ethiopia 2000-2020: A systematic review and meta-analysis
Source: PLoS Negl Trop Dis. 2021 Sep 15;15(9):e0009781. doi: 10.1371/journal.pntd.0009781 (PMC8476039; doi:10.1371/journal.pntd.0009781)

**S1_ Fig. Boxplot of studies on prevalence of *P. vivax* infection in Ethiopia**

**
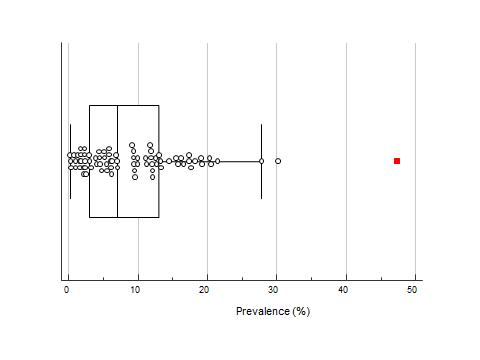
**

Supplement: S1 Fig — (DOCX) [file pntd.0009781.s004.docx]

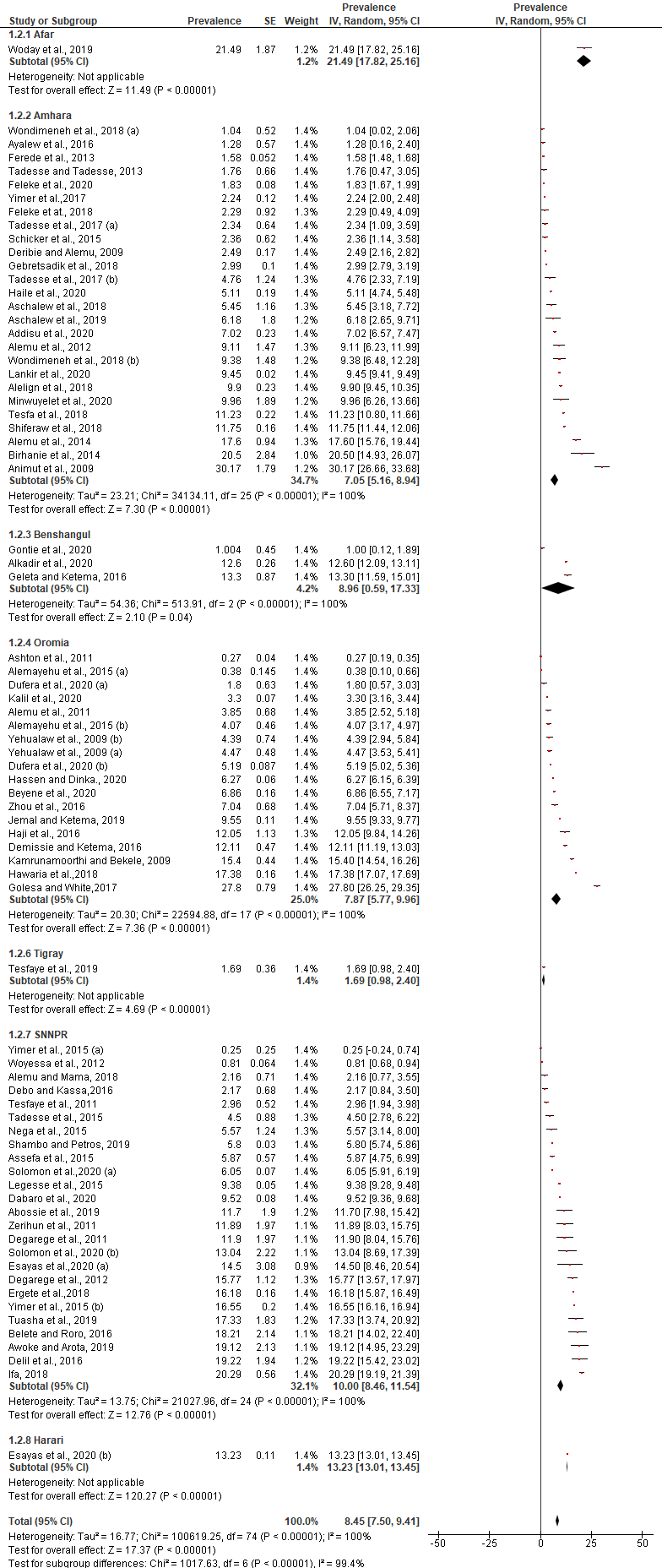


**S3_Fig.** Pooled estimates of prevalence of *P. vivax* for different locations/regions of Ethiopia

Supplement: S3 Fig — (DOCX) [file pntd.0009781.s006.docx]

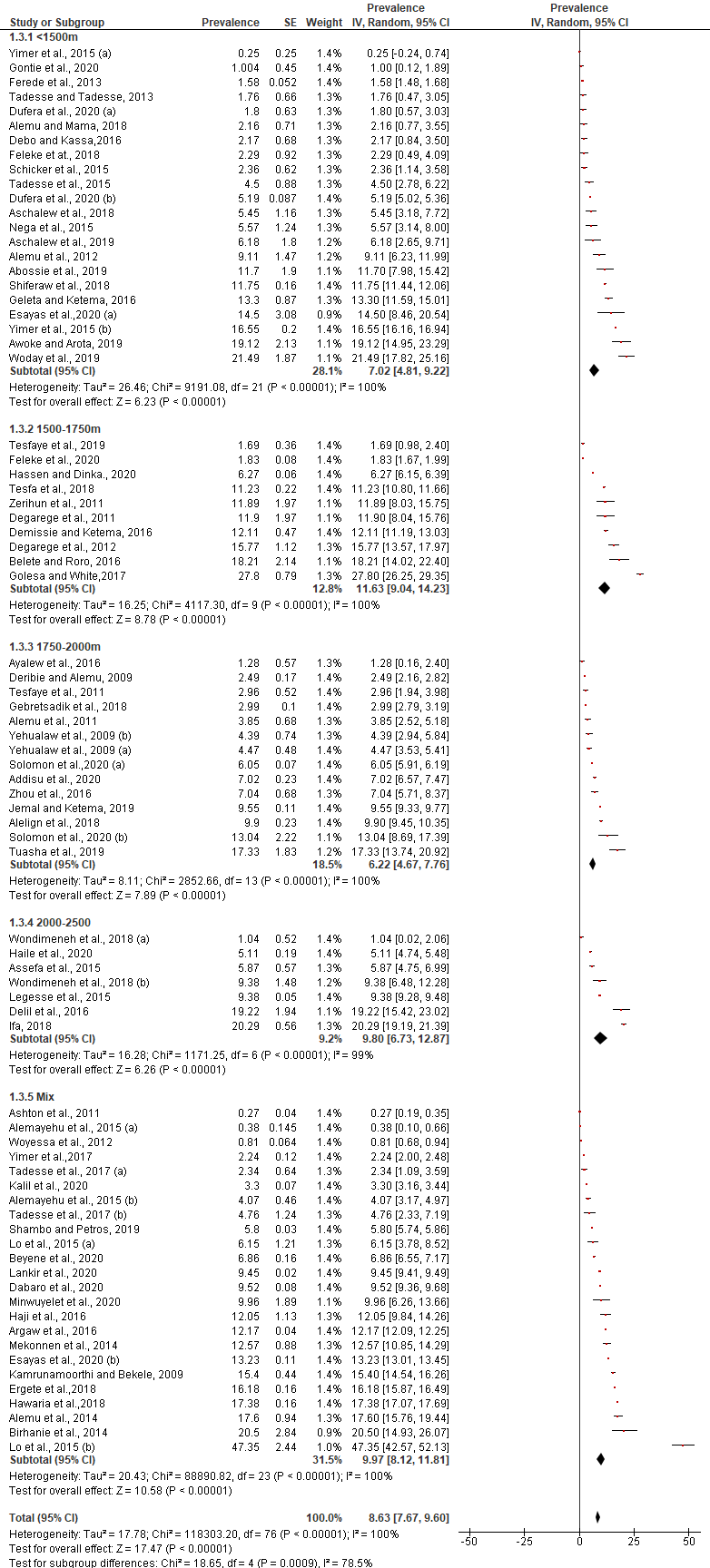


**S4_Fig.** Estimate prevalence of P. vivax in different eco-epidemiological zones of Ethiopia

Supplement: S4 Fig — (DOCX) [file pntd.0009781.s007.docx]

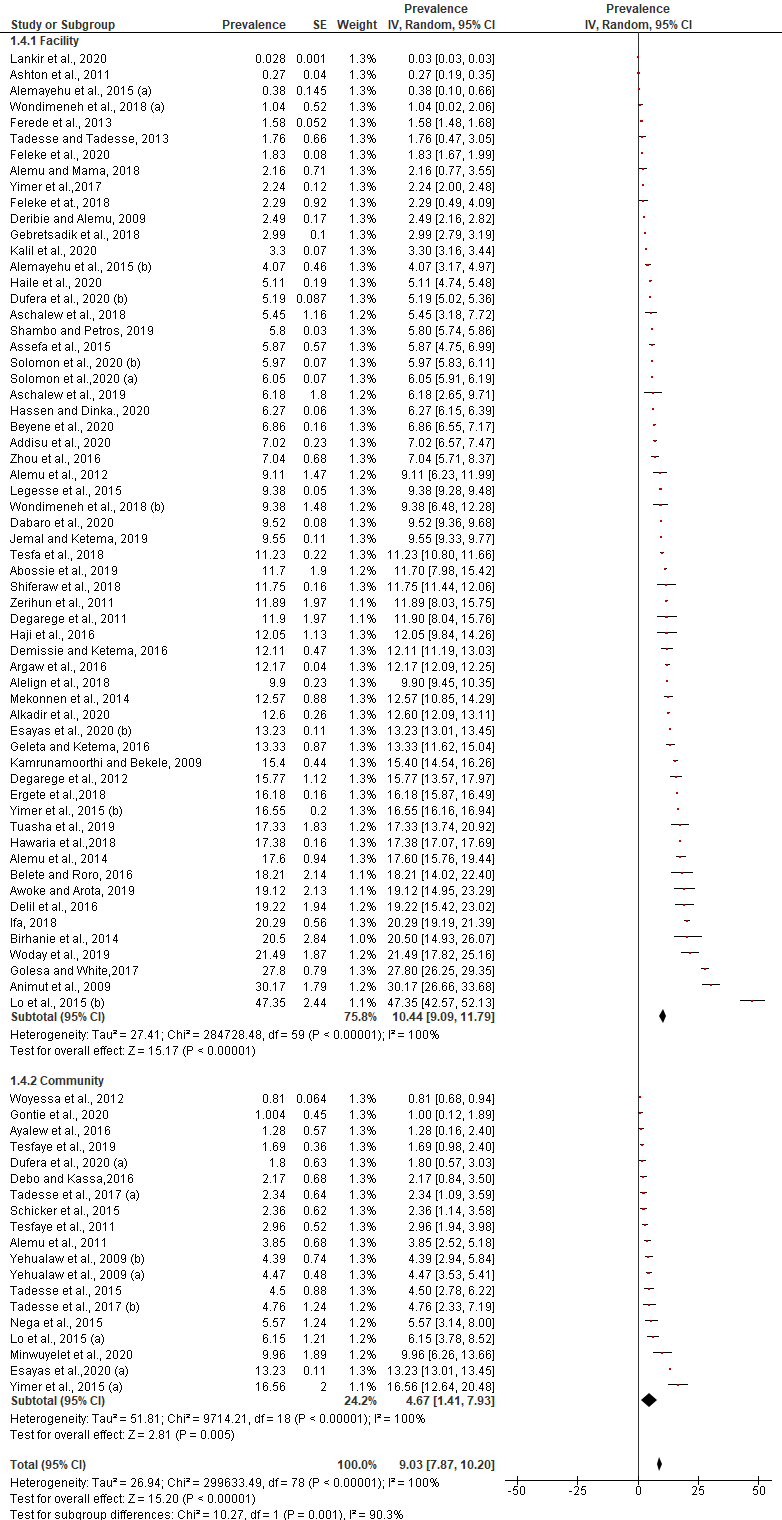


**S5_Fig.** Prevalence of *P. vivax* at different study settings in Ethiopia

Supplement: S5 Fig — (DOCX) [file pntd.0009781.s008.docx]

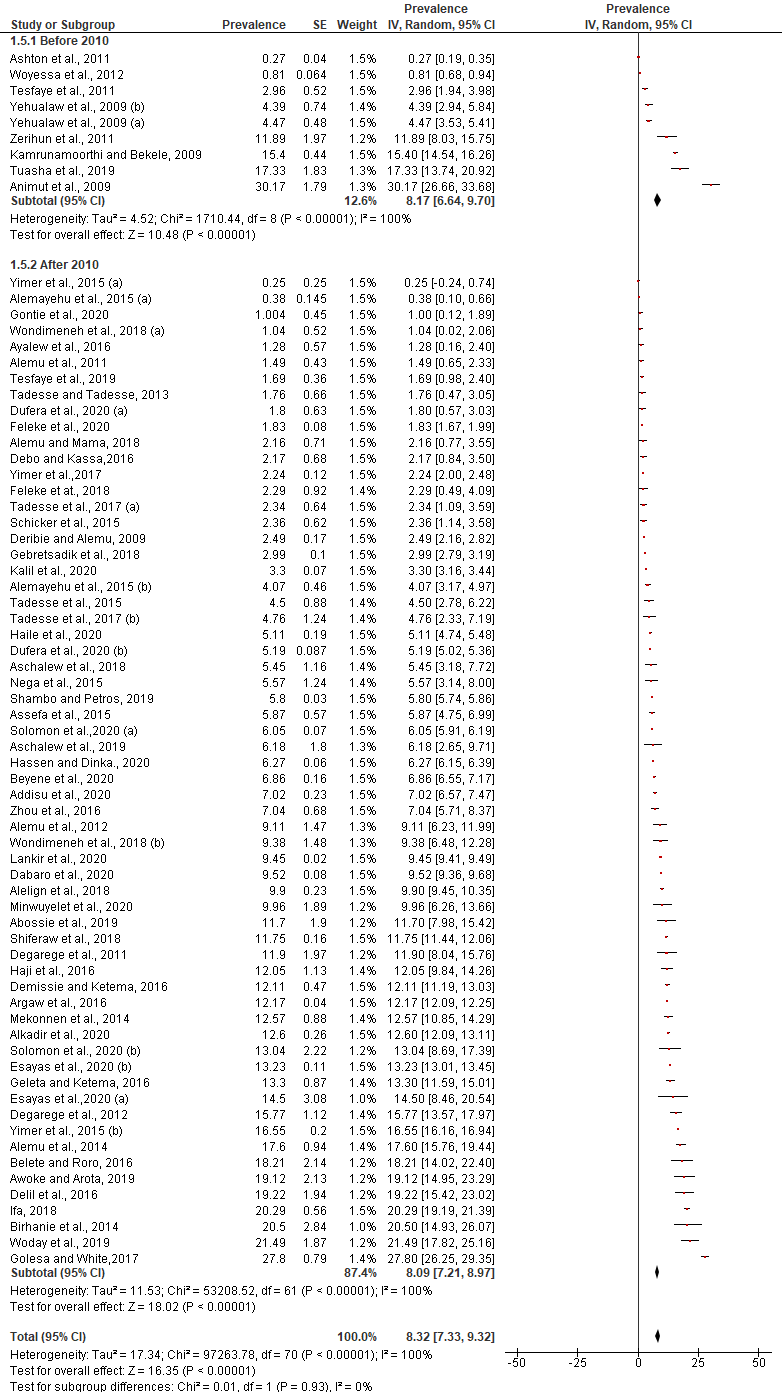


**S6_Fig.** Prevalence of *P. vivax* with respect to year of survey in Ethiopia

Supplement: S6 Fig — (DOCX) [file pntd.0009781.s009.docx]
