# Supplementary material for: Plasmodium vivax epidemiology in Ethiopia 2000-2020: A systematic review and meta-analysis
Source: PLoS Negl Trop Dis. 2021 Sep 15;15(9):e0009781. doi: 10.1371/journal.pntd.0009781 (PMC8476039; doi:10.1371/journal.pntd.0009781)

**S2_Fig.** Funnel plot for publication bias assessment of studies on prevalence of *P. vivax* infection in Ethiopia


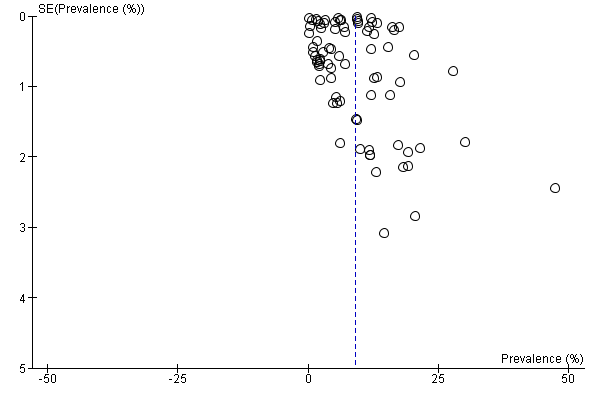

Supplement: S2 Fig — (DOCX) [file pntd.0009781.s005.docx]
