# Supplementary material for: Plasmodium vivax epidemiology in Ethiopia 2000-2020: A systematic review and meta-analysis
Source: PLoS Negl Trop Dis. 2021 Sep 15;15(9):e0009781. doi: 10.1371/journal.pntd.0009781 (PMC8476039; doi:10.1371/journal.pntd.0009781)
Supplement: Attachment — Submitted filename: Response to reviewers comment_ Plos.Neg. Trop.Dis.docx [file pntd.0009781.s010.docx]

To the Associate Editor
PLOS Neglected Tropical Diseases

30^th^ July, 2021

Dear Dr Paul O. Mireji,

Manuscript: PNTD-D-21-00796) - [EMID: 7f3f20a977cccc9d] **Plasmodium vivax   epidemiology in Ethiopia 2000-2020: a systematic review and meta-analysis.**

Thank you for the opportunity to revise and resubmit our manuscript. The reviewers gave positive and constructive feedback. We have responded to their comments and provide detailed responses below. We believe that the revised manuscript addresses all of the concerns and suggestions raised and is improved significantly.

We hope that the manuscript is now acceptable for publication.

Sincerely,

**Prof. Tsige Ketema Mamo**

**Editorial comments**

Figure Files:
While revising your submission, please upload your figure files to the Preflight Analysis and Conversion Engine (PACE) digital diagnostic tool, [https://pacev2.apexcovantage.com](https://pacev2.apexcovantage.com/). PACE helps ensure that figures meet PLOS requirements. To use PACE, you must first register as a user. Then, login and navigate to the UPLOAD tab, where you will find detailed instructions on how to use the tool. If you encounter any issues or have any questions when using PACE, please email us at [figures@plos.org](mailto:figures@plos.org).

**Response:** We have uploaded the figures as separate files in the correct order.

Data Requirements:
Please note that, as a condition of publication, PLOS' data policy requires that you make available all data used to draw the conclusions outlined in your manuscript. Data must be deposited in an appropriate repository, included within the body of the manuscript, or uploaded as supporting information. This includes all numerical values that were used to generate graphs, histograms etc.. For an example see here: <http://www.plosbiology.org/article/info%3Adoi%2F10.1371%2Fjournal.pbio.1001908#s5>.

**Response:** the data requirement policy is not applicable for our study

Reproducibility:
To enhance the reproducibility of your results, we recommend that you deposit your laboratory protocols in [protocols.io](http://protocols.io/), where a protocol can be assigned its own identifier (DOI) such that it can be cited independently in the future. Additionally, PLOS ONE offers an option to publish peer-reviewed clinical study protocols. Read more information on sharing protocols at <https://plos.org/protocols?utm_medium=editorial-email&utm_source=authorletters&utm_campaign=protocols>

**Response:** laboratory protocols are not applicable for our study

References
Please review your reference list to ensure that it is complete and correct. If you have cited papers that have been retracted, please include the rationale for doing so in the manuscript text, or remove these references and replace them with relevant current references. Any changes to the reference list should be mentioned in the rebuttal letter that accompanies your revised manuscript. If you need to cite a retracted article, indicate the article's retracted status in the References list and also include a citation and full reference for the retraction notice.

**Response:** All references are checked for their completeness and correctness.

**Response to the reviewers’ comment**

**Reviewer #1:**

1. The paper would benefit by including a sketch map showing regions of high and low prevalence and also areas where *P. vivax* is expanding in Ethiopia.

**Response:** Thank you for this suggestion. We tried to include the prevalence level indicators in the map. But because of variability of reports from the same district/region, which was varied in terms of years too, it was difficult to sketch a map showing this vital information. In the revised map we tried to add a color degradation code to make identification of the different prevalence easier (e.g. from very light pink to dark pink) to differentiate those overlapping too (Figure 2).

2. Plasmodium vivax is a serious public health problem in Ethiopia and neighboring countries….
   Nevertheless they may make reference to the potential effect of changes in vector ecology and in particular to environmental change. Vectors such as Anopheles arabiensis, Anopheles coustani and Anopheles pharoensis are exophilic and partially anthropophagic making their control difficult. The authors may wish to address this issue in the discussion.

**Response: Response:** We agree that it is important to include this information. We have revised the discussion part and change is highlighted yellow.

**Reviewer #2**:

3. Yes the conclusions are supported by the results and discussions are important in the field of vivax malaria epidemiology.
   The only error observed was for some reference e.g. ref 95 Kibret et al., 2014 that does not discuss vivax per se but rather the malaria vector. It is important that all references are correctly cited

**Response:** Thank you for detecting this type error. It was supposed to be reference # 94 not 95. Now corrected.
