# Supplementary material for: Plasmodium vivax epidemiology in Ethiopia 2000-2020: A systematic review and meta-analysis
Source: PLoS Negl Trop Dis. 2021 Sep 15;15(9):e0009781. doi: 10.1371/journal.pntd.0009781 (PMC8476039; doi:10.1371/journal.pntd.0009781)
Supplement: Attachment — Submitted filename: P vivax Review Remarks II AKG.docx [file pntd.0009781.s011.docx]

**Review Editor**

**Review remarks**

The systematic review study of the prevalence of *Plasmodium vivax* in Ethiopia has been undertaken in accordance with the rules of such a review. The selection of the review material was undertaken in a well set out criteria of inclusion and exclusion to avoid bias. The conclusions of the review are based on evidence derived from carefully selected publications.

The manuscript is well written and has no language issues.

The manuscript could however benefit by adding a sketch map delineating areas of high and low prevalence of *P. vivax* in Ethiopia.

The authors should postulate/hypothesis the driving factors of the geographic expansion of *P. viva*x in Ethiopia. Why is the disease shifting west-wards ?. What is the role of *Anopheles coustani* and *Anopheles pharoensis* compared to *Anopheles arabiensis* in the transmission of the disease ? Could vector ecology be affection the observed disease range shift ?

**Review Editor comments**

The authors have responded to the above comments adequately. The manuscript is now ready for publication.
